# Feature-Based Growth Curve Classification Enables Efficient Phage Discrimination

**DOI:** 10.3390/v18010092

**Published:** 2026-01-09

**Authors:** Yuma Oka, Keidai Miyakawa, Moe Yamazaki, Yuki Maruyama

**Affiliations:** H.U. Group Research Institute G.K., Akiruno 197-0833, Tokyo, Japan; keidai.miyakawa@hugp.com (K.M.); moe.yamazaki@hugp.com (M.Y.); yuki.maruyama@hugp.com (Y.M.)

**Keywords:** bacteriophage screening, growth curve analysis, unsupervised clustering, phage therapy

## Abstract

Rapid isolation of therapeutic bacteriophages from environmental sources is essential for personalized phage therapy, particularly when appropriate phages are unavailable in existing banks. However, comprehensive characterization of all candidate phages is resource-intensive, especially when plaque morphologies are similar and fail to discriminate between distinct phages. Here, we present an upstream screening approach that utilizes co-culture growth curve analysis to rapidly triage phage isolates during the early isolation process. We extracted seven biologically meaningful features that capture lysis kinetics, lysis efficiency, and post-lysis dynamics from bacterial growth curves and applied unsupervised clustering algorithms for phage discrimination. Validation using T-phages at a multiplicity of infection of 0.01 demonstrated superior clustering performance (Adjusted Rand Index = 0.881 ± 0.057) compared to established metrics including the Virulence Index and Centroid Index. Application to phages isolated from sewage successfully identified all three genomically distinct species present (sampling score = 1.0), enabling targeted selection of representative phages for downstream characterization. This approach reduced candidates requiring detailed analysis by two-thirds (from 21 to 7 isolates) while maintaining complete species coverage, thereby providing an efficient and scalable screening tool that reduces workload for downstream analyses and accelerates discovery of novel therapeutic phages for clinical applications.

## 1. Introduction

Antimicrobial resistance (AMR) is a growing global threat. A recent systematic analysis projected that by 2050, the annual number of deaths attributable to AMR will reach 1.91 million, while annual deaths associated with AMR are expected to total 8.22 million [1]. To combat AMR, bacteriophages (or phages) have recently gained increasing attention as a promising alternative to antibiotics, as phages kill bacteria through mechanisms distinct from those of traditional antibiotics. Phage therapy, which involves the use of phages to treat bacterial infections, has been increasingly applied in clinical settings, with a growing number of clinical studies supporting its feasibility and efficacy [2,3,4,5].

Phages specifically infect bacteria and do not affect other organisms. This makes phage therapy a highly targeted approach that minimally impacts the human microbiome, as demonstrated in both human and animal studies [6,7,8]. To leverage these advantages, phage banks have been established or are under development worldwide to collect, store, and characterize therapeutic phages [9,10,11,12]. When a bacterial pathogen is identified, phages in these phage banks are screened to find suitable candidates for treatment. However, due to the vast diversity of bacterial strains and the host specificity of phages, it is impractical to maintain a comprehensive repository containing all effective phages. In cases where no suitable phage is available, the isolation of new phages from environmental sources becomes essential [13,14].

New phages are traditionally isolated using plaque assays, which involve co-culturing host bacteria with phage-containing samples on agar plates [15,16,17]. While widely used, this method is time-consuming and labor-intensive. Moreover, when multiple phages with similar plaque morphology are present in a single sample, distinguishing between them becomes challenging. Accurate identification often requires additional analyses such as transmission electron microscopy, host range profiling, and genomic sequencing. These procedures, while informative, are resource-intensive and hinder the rapid isolation of diverse phage strains. Therefore, there is a pressing need for more efficient and scalable phage isolation technologies to support clinical applications [18].

Here, we propose and validate the use of growth curve data obtained from co-culturing phages with host bacteria as a tool for phage discrimination. These curves reflect the dynamics of bacterial lysis and can reveal subtle differences in phage behavior that are not apparent in plaque morphology. While computational methods have been applied to classify lytic activity from such data [19,20,21,22,23], these applications have primarily focused on post-isolation characterization. The potential of growth curve analysis as a tool to distinguish and triage multiple phages directly from a mixed sample during the isolation process remains largely unexplored. This study introduces a novel approach that extracts features from growth curves and applies clustering techniques to discriminate distinct phage strains as an upstream screening step, prior to detailed analyses. By implementing this screening at an early stage, we can strategically prioritize which phage candidates require resource-intensive downstream analyses such as genomic sequencing, thereby offering the potential to significantly reduce both the time and cost required to discover novel therapeutic phages. We hypothesize that this approach will enable more efficient isolation and discrimination of multiple phage strains from environmental samples. This advancement could significantly accelerate the development of phage therapeutics.

## 2. Materials and Methods

### 2.1. T-Phages

T-phages (T1–T7) were obtained from Biological Resource Center, National Institute of Technology and Evaluation, Japan (NBRC). They were maintained at 4 °C in SM buffer (50 mM Tris-HCl, pH 7.5; 100 mM NaCl; 8 mM MgSO_4_; 0.01% gelatin). Phage titers were determined by double-layer plaque assays.

### 2.2. Bacterial Host Strains

*Escherichia coli* strain NBRC13168 (B strain) was used as the host for T-phage experiments because B strains are widely used hosts for T-phages. NBRC13898 (C strain) was used for environmental phage isolation because it showed high phage isolation efficiency in preliminary experiments. Both strains were cultured in Luria–Bertani (LB) broth (Becton, Dickinson and Company, Franklin Lakes, NJ, USA) at 37 °C with shaking at 200 rpm until the optical density at 600 nm (OD_600_) reached approximately 0.5.

### 2.3. Phage Isolation from an Environmental Source

Environmental phages were isolated from sewage collected using an absorbent cotton sampler suspended overnight in a building sewage outlet. The collected sewage was centrifuged at 8000× *g* for 5 min to remove debris, and the supernatant was filtered through a 0.22 µm polyvinylidene difluoride (PVDF) membrane (Merck Millipore, Burlington, MA, USA). The filtrate was mixed with *E. coli* NBRC13898 culture in melted 0.5% LB soft agar and overlaid onto LB agar plates. Plates were incubated overnight at 37 °C. The resulting plaques were picked with sterile pipette tips and resuspended in LB broth.

### 2.4. Plaque Imaging

Plaque morphologies were photographed using an iPhone SE (2nd generation; Apple).

### 2.5. Quantification of Growth Curves

#### 2.5.1. MOI-Controlled Experiments with T-Phages

Growth dynamics were monitored using a Varioskan LUX microplate reader (Thermo Fisher Scientific, Waltham, MA, USA). Mid-exponential phase cultures (OD_600_ = 0.5) were diluted 10-fold in fresh LB broth and dispensed into 96-well plates. T-phages were added to achieve a final multiplicity of infection (MOI) of 0.01, 0.1, or 1.0, calculated based on the bacterial concentration at the time of infection. Plates were incubated at 37 °C with continuous orbital shaking (600 rpm) for 24 h, with OD_600_ measurements recorded every 10 min. Three biological replicates were performed for each condition to verify the consistency of cluster assignment among samples from the same phage species.

#### 2.5.2. Plaque-Derived Phage Experiments

To simulate practical scenarios where precise titering is unavailable, we conducted an experiment using freshly harvested phages from plaques. Individual plaques from each T-phage were picked with sterile pipette tips, resuspended in 1 mL LB broth, and filtered through 0.22 µm PVDF membranes. Without titer determination, 1 µL of each filtrate was added to 99 µL of bacterial cultures prepared as described above. One out of three T6 phage samples did not show any lysis at all, so it was excluded from further analysis. This approach mimics the workflow for environmental phage characterization where immediate classification is desired without extensive preliminary titering.

#### 2.5.3. Environmental Phage Characterization

Twenty-four isolated environmental phages were tested using the same growth curve protocol as the plaque-derived T-phage experiments. Sample 2 failed to amplify sufficiently, and adequate genomic DNA could not be extracted. Samples 16 and 21 did not show any lysis at all. These samples were therefore excluded from further analysis. The remaining twenty-one isolates were included in subsequent analysis.

### 2.6. Data Processing and Feature Extraction

#### 2.6.1. Growth Curve Preprocessing

Raw OD_600_ measurements were processed using Python v3.12.2 through the following pipeline: (i) blank subtraction using media-only control wells, (ii) application of a Savitzky–Golay filter implemented in SciPy v1.15.1 using scipy.signal.savgol_filter (window length = 3, polynomial order = 1) to reduce measurement noise while preserving curve dynamics, and (iii) time alignment by setting t = 0 at the minimum OD observed in uninfected controls. To ensure all values remained positive for subsequent calculations, a constant offset was added to all measurements. If any negative values were present after blank subtraction, this offset was defined as the absolute value of the minimum OD reading observed across the entire dataset.

#### 2.6.2. Feature Extraction (GC7 Feature Set)

From each processed growth curve, we extracted seven quantitative features, collectively designated as the GC7 feature set, designed to comprehensively capture the dynamics of phage-mediated bacterial lysis (Figure S1a). All feature extraction was implemented in Python using NumPy v1.26.4 [24] and SciPy v1.15.1 [25]:

• Peak count was determined using the scipy.signal.find_peaks function with minimum prominence threshold exceeding 0.01, window_size = 30, and plateau_size = 1;
• Drop slope was calculated as the linear regression slope of OD_600_ values from the peak to the bottom using scipy.stats.linregress, representing the average rate of bacterial killing during the lysis phase;
• Drop magnitude represented the difference between the peak OD and the bottom OD, indicating the total bacterial biomass eliminated;
• OD at bottom was identified as the OD_600_ value at the point after the first peak where the instantaneous slope (calculated using numpy.gradient) had diminished to 10% of the maximum negative slope observed during the lysis phase;
• Time from peak to bottom (Time (peak-bottom)) measured the duration from peak to bottom;
• Time from bottom to OD regrowth (Time (bottom-OD rise)) was defined as the duration from OD at bottom to the point where OD increased to 110% of bottom OD;
• Post-lysis area under curve (AUC (bottom-end)) was calculated as the integrated area from the bottom point to the experiment end using the trapezoidal rule implemented in scipy.integrate.trapezoid.

We performed sensitivity analysis for the OD at bottom threshold parameter, comparing the default value (10% of maximum lysis rate) with alternative thresholds (5% and 15%). Leave-One-Species-Out cross-validation (LOSOCV) was performed using GC7 features with K-means clustering on MOI 0.01 data. Clustering performance remained stable across all threshold values (Figure S1b–e), confirming the robustness of our threshold selection.

Prior to clustering analysis, all seven features were standardized to zero mean and unit variance using the StandardScaler function from scikit-learn v1.6.1 [26], ensuring that features with different scales contributed equally to the clustering process. The complete analysis code, including preprocessing, feature extraction, and clustering scripts, is provided as Supplementary Code, with detailed documentation for replication and adaptation.

### 2.7. Clustering Analysis

#### 2.7.1. Algorithm Implementation

We evaluated four distinct clustering algorithms, each based on different mathematical assumptions about data structure, to ensure robust classification independent of algorithmic choice. For K-means clustering, which assumes spherical clusters of similar size, we determined the optimal number of clusters by comparing results from two complementary methods. The elbow method identified the point of maximum curvature in the within-cluster sum of squares plot, while silhouette analysis determined the cluster number that maximized the mean silhouette coefficient. When these methods suggested different values, we selected the larger to avoid potential under-clustering that might merge biologically distinct phage species.

Gaussian Mixture Model (GMM) clustering, which can accommodate elliptical clusters with varying sizes and orientations, was optimized by minimizing the Bayesian Information Criterion (BIC) across models. We employed full covariance matrices, allowing the algorithm to capture complex cluster shapes that might reflect the continuous variation in phage phenotypes.

For Density-Based Spatial Clustering of Applications with Noise (DBSCAN), we estimated the critical epsilon parameter using k-distance graphs with k = 2 neighbors. The epsilon value was selected at the “elbow” point where the k-nearest-neighbor distances showed maximum curvature, representing the natural density threshold in the data. We set min_samples = 1 to ensure complete classification of all isolates, as our goal was to characterize every phage rather than identify core clusters, which differs from typical outlier-detection applications of DBSCAN.

Hierarchical clustering was performed using Ward’s linkage method, which minimizes within-cluster variance at each merging step. The optimal number of clusters was determined by evaluating both silhouette scores and Davies–Bouldin indices across different dendrogram cut heights, selecting the configuration that maximized cluster separation while maintaining internal cohesion.

#### 2.7.2. Cross-Validation Strategy

To rigorously assess the robustness of our classification framework, we implemented LOSOCV. In each iteration, one of the seven T-phage species was systematically excluded from the dataset, and clustering was performed on the remaining six species. The optimal number of clusters for each clustering algorithm was re-determined in each iteration using the same criteria as described above. This approach tested whether the feature space structure and clustering patterns remained stable despite variations in phage composition, effectively simulating real-world scenarios where the diversity of phages in a sample cannot be predetermined. The consistency of clustering performance across these iterations would indicate that our features capture fundamental aspects of phage biology rather than dataset-specific patterns.

#### 2.7.3. Performance Evaluation

We assessed clustering performance using three complementary metrics that capture different aspects of classification quality. The Adjusted Rand Index (ARI) measured the agreement between cluster assignments and true species labels while correcting for agreements occurring by chance, with values approaching 1.0 indicating near-perfect clustering. Normalized Mutual Information (NMI) quantified the information shared between the clustering results and true labels, normalized to range from 0 to 1, where higher values indicated better preservation of species identity information in the clusters. Additionally, we calculated a sampling score that represented the probability of recovering all phage species when selecting one representative isolate from each cluster, directly addressing the practical utility of clustering for phage collection management and diversity assessment (see Supplementary Methods for the mathematical formulation). Importantly, ground truth labels representing phage species identity were used exclusively for post hoc performance evaluation and were never included in the clustering process itself, maintaining the unsupervised nature of our classification approach.

#### 2.7.4. Comparative Analysis with Established Methods

To contextualize the performance of our GC7 feature set, we conducted comprehensive comparisons with previously published phage characterization metrics: the Virulence Index (VI) [19], the Centroid Index (CI) [20], and non-metric multidimensional scaling (NMDS) of complete growth curves [21]. These methods represent diverse analytical approaches to growth curve classification, encompassing one-dimensional summary metrics (VI and CI) and multivariate ordination (NMDS). VI was calculated by integrating local virulence. CI was computed as the normalized shift in growth curve centroids. For NMDS analysis, distance matrices were calculated as the sum of absolute differences in OD_600_ at each time point between all sample pairs and projected into two-dimensional space using the metaMDS function from the vegan package v2.7.1 in R v4.5.1.

### 2.8. Genomic DNA Extraction and Sequencing

Phage genomic DNA was extracted using phenol–chloroform extraction. Phage suspensions were incubated overnight at 4 °C in a solution containing 4% (*w/v*) PEG 8000 and 500 mM NaCl, followed by centrifugation at 10,000× *g* for 30 min. The resulting pellet was resuspended in SM buffer and treated with 2 U TURBO DNase (Thermo Fisher Scientific) and 10 µg RNase A (Nippon Gene, Tokyo, Japan) at 37 °C for 30 min. Phenol/chloroform/isoamyl alcohol (25:24:1, Nacalai Tesque, Kyoto, Japan) extraction was performed twice, followed by chloroform extraction. DNA was precipitated with 3 M sodium acetate and isopropanol, centrifuged at 15,000× *g* for 15 min, washed with 70% ethanol, and resuspended in TE buffer. For samples 11, 12, 14, and 23, library preparation was conducted using the Rapid Sequencing DNA V14 Barcoding Kit (SQK-RBK114.96, Oxford Nanopore Technologies, Oxford, UK), and sequencing was performed on a MinION Mk1C device using an R10.4.1 Flongle flow cell (Oxford Nanopore Technologies). For the remaining samples, library preparation was conducted using the NEBNext Ultra II FS DNA Library Prep Kit for Illumina (New England Biolabs, Ipswich, MA, USA), and sequencing was performed on MiSeq using MiSeq Reagent Nano Kit v2 (500 Cycles; Illumina, San Diego, CA, USA). The choice of sequencing platform was based on the DNA yield obtained from each sample.

### 2.9. Genome Analysis

The sequenced data from Flongle sequencing were basecalled into reads and demultiplexed with Dorado v1.0.2 with sup mode. Reads were filtered using Chopper v0.8.0 (quality threshold is over 10) [27] and assembled with Flye v2.9.6 [28]. The sequenced data from MiSeq sequencing were filtered using fastp v1.0.1 [29,30,31] with the default options and assembled with SPAdes v4.2.0 [32] with the following option (—isolate). Average Nucleotide Identity (ANI) was compared with ANIm in pyani-plus v1.0.0 with the default options [33].

### 2.10. Statistical Analysis

Inter-species differences in individual features were assessed using the Kruskal–Wallis test with Holm correction. Multivariate growth curve data were analyzed using permutational multivariate analysis of variance (PERMANOVA) with 9999 permutations using the adonis2 function in the vegan package, with homogeneity of multivariate dispersions verified using the permutest function in the vegan package. All statistical analyses were performed in R v4.5.1.

## 3. Results

### 3.1. Development of Growth Curve-Based Phage Classification

To establish a quantitative framework for phage classification, we analyzed bacterial growth curves from controlled infection experiments using seven canonical T-phages (T1–T7) as a model system. These phages were selected for their well-characterized biology and diverse lytic behaviors, providing a suitable model system for our approach. Among these, T2, T4, and T6 belong to the same genus and exhibit similar plaque morphologies (Figure S2a,b; raw images: Supplementary Data). T3, T5, and T7 also display similar plaque appearances, although T5 belongs to a different taxonomic group. Genomic analysis revealed ANI values exceeding 0.9 between T3 and T7, and among T2, T4, and T6 (Figure S2c). These morphological similarities across both related and unrelated phages demonstrate the challenge of visual discrimination and support the need for growth curve-based classification.

From the resulting growth curves, we extracted seven quantitative features organized into three biologically meaningful categories. First, lysis kinetics parameters captured the temporal aspects of infection: duration of the primary lysis phase (Time (peak-bottom)) and average lysis rate (Drop slope). Second, lysis efficiency metrics quantified the extent of bacterial killing: the magnitude of optical density drop (Drop magnitude) and minimum OD reached (OD at bottom). Third, post-lysis dynamics described bacterial population recovery: the number of regrowth peaks (peak count), time to first regrowth (Time (bottom-OD rise)), and integrated area under the curve following initial lysis (AUC (bottom-end)). These features were selected to comprehensively capture the diversity of phage–host interaction outcomes, from rapid complete lysis to partial killing with resistant population emergence.

### 3.2. Feature Characteristics and Distribution

**Figure 1:**
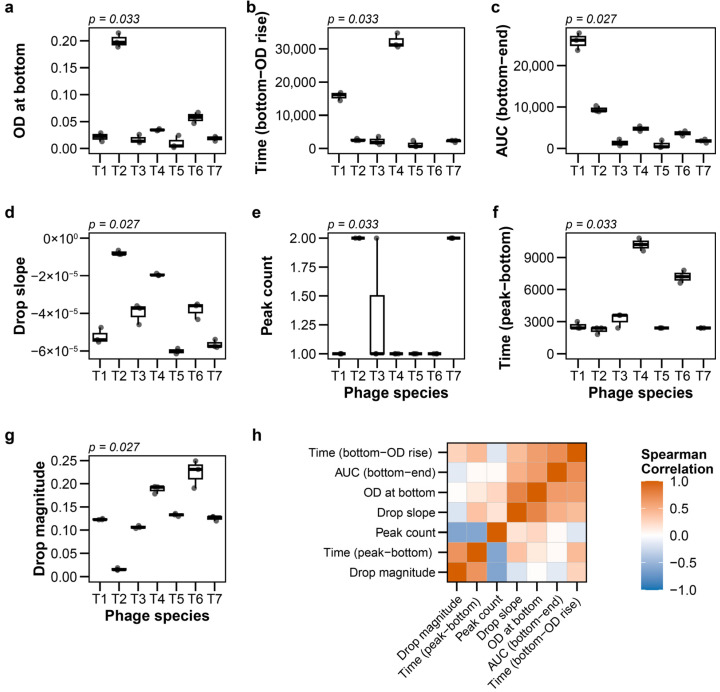
Feature distributions and correlations for T-phages at multiplicity of infection (MOI) 0.01. (**a**–**g**) Boxplots showing distributions of seven features among T-phage species (*n* = 3 for individual species): (**a**) OD at bottom, (**b**) Time to OD regrowth from bottom, (**c**) Post-lysis AUC (bottom to endpoint), (**d**) Drop slope, (**e**) Peak count, (**f**) Time from peak to bottom, (**g**) Drop magnitude. *p* values from Kruskal–Wallis tests corrected by Holm are displayed above each panel. (**h**) Pairwise Spearman correlations among all features.

At MOI 0.01, the seven extracted features showed significantly different distributions among phage species (all *p* < 0.05, Kruskal–Wallis test with Holm correction; Figure 1a–g; raw data: Table S5). For instance, the drop slope, which is one of the lysis kinetics parameters, demonstrated differences among species (*p* = 0.027).

Analysis of feature relationships revealed that each metric captured distinct aspects of the lysis process. All pairwise Spearman correlation coefficients between features were below 0.8, with most showing moderate to low correlation (Figure 1h). Notably, different features distinguished different species pairs (Figure 1a–g). For example, T1 and T3 phages exhibited similar OD at bottom but differed markedly in Time (bottom-OD rise), while other pairs showed distinct patterns of feature-specific separation. This indicates that no single feature alone captures the full diversity of phage lytic behaviors. The seven features thus provide complementary, rather than redundant, information about phage–host interactions. The combination of temporal (kinetics), quantitative (efficiency), and recovery (post-lysis) parameters provides a comprehensive characterization of phage lytic behavior.

Comparison with established analytical approaches confirmed the validity of studying growth curve features for phage classification. Established metrics, specifically the VI (*p* = 0.005) and CI (*p* = 0.006), also showed significant inter-species variation (Figure S3a,b). Similarly, multivariate analysis of complete growth curve coordinates using NMDS, which is another established approach, revealed clear species-specific clustering (PERMANOVA *p* = 1 × 10^−4^, PERMDISP *p* = 0.3213; Figure S3c). These results from multiple independent analytical methods consistently demonstrate that T-phages produce distinct and characteristic growth curve signatures, supporting the feasibility of phage classification based on growth curves.

### 3.3. Validation of Classification Framework Using Controlled Phage Infections

To evaluate the practical utility of our classification approach based on growth curves, we performed systematic validation experiments using T1–T7 phages at predetermined titers. The set of seven features, referred to as GC7 in this study, was extracted from growth curves measured at an MOI of 0.01. This feature set was then evaluated using several clustering algorithms, each based on different underlying assumptions. These included partitional (K-means), probabilistic (GMM), density-based (DBSCAN), and hierarchical methods. This approach ensured robust performance assessment independent of algorithmic choice.

#### 3.3.1. Leave-One-Species-Out Cross-Validation at MOI 0.01

To assess the robustness of our classification framework, we performed LOSOCV, systematically excluding each of the seven phage species and evaluating classification performance on the remaining six species. This approach models realistic situations in which environmental or clinical samples may contain unknown mixtures of phage species. It allows assessment of whether the unsupervised clustering method can accurately determine both the number and identity of species present, irrespective of the sample composition.

**Figure 2:**
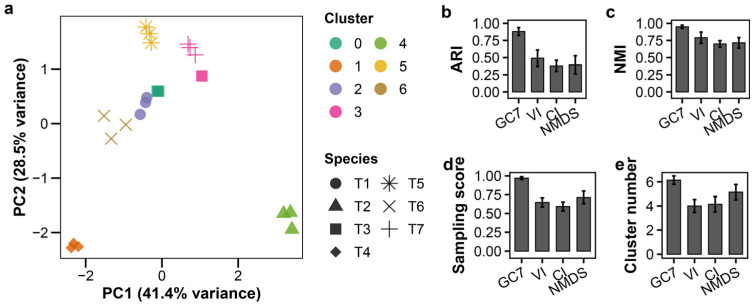
Growth curve-based classification of T-phages at MOI 0.01 and comparison with established methods. K-means clustering of seven T-phage species (T1–T7; *n* = 3 per species) using GC7 features extracted from growth curves at MOI 0.01. (**a**) PCA plot of clustering results; colors indicate clusters, shapes indicate species. (**b**–**e**) Leave-One-Species-Out cross-validation (LOSOCV) performance comparing GC7 with established methods (Virulence Index (VI), Centroid Index (CI), NMDS coordinates (NMDS)): (**b**) Adjusted Rand Index (ARI), (**c**) Normalized Mutual Information (NMI), (**d**) sampling score, (**e**) cluster count. Error bars indicate standard deviation.

K-means clustering with GC7 showed clear distinction among all seven T-phage species at MOI 0.01 (Figure 2a). In the LOSOCV analysis, GC7 achieved an Adjusted Rand Index (ARI) of 0.881 ± 0.057 (mean ± standard deviation), indicating strong agreement with true species labels (Figure 2b; Table S1). This performance was confirmed by high Normalized Mutual Information (NMI) and sampling scores, with the algorithm correctly identifying approximately six clusters (Figure 2c–e).

To benchmark the performance of GC7, we compared these results with established analytical features under the same LOSOCV framework. GC7 substantially outperformed VI, CI, and two-dimensional NMDS coordinates across all key metrics (Figure 2b–e; Table S1). These previously established features yielded significantly lower ARI and NMI values. Notably, their lower sampling scores and tendency to underestimate the number of clusters (4.0–5.1) revealed a limited ability to recover all species present.

The superior performance of GC7 can be attributed to its multi-dimensional structure. While VI and CI effectively compress temporal dynamics into single scalar values, they necessarily lose information about distinct infection phases. NMDS preserves rank-order relationships but projects data into two dimensions, potentially discarding subtle variation. In contrast, GC7’s seven-feature design maintains low correlation structure (r < 0.8), ensuring each dimension contributes distinct information. This structured dimensionality enables more accurate classification than either single-metric or dimension-reduced approaches.

Alternative clustering algorithms confirmed the robustness of GC7 (Figure S4; Table S1). GMM also achieved high performance (ARI = 0.809 ± 0.130), identifying 5.4 ± 0.5 clusters on average. This suggests GMM occasionally merged species with similar lytic profiles. DBSCAN showed comparable performance (ARI = 0.797 ± 0.146) but with higher variance in cluster numbers. In contrast, hierarchical clustering yielded consistently lower performance metrics across all feature sets.

Together, these results demonstrate that the GC7 feature set provides a robust foundation for accurate, unsupervised classification, with K-means offering the most stable performance under these controlled conditions

#### 3.3.2. Performance Across Multiple MOI Conditions

**Figure 3:**
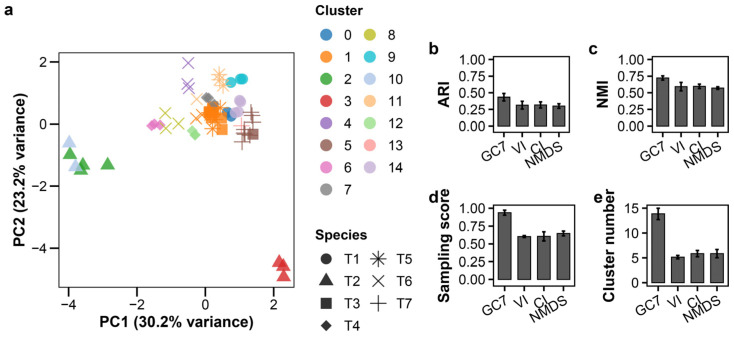
Growth curve-based classification of T-phages at multiple MOIs. K-means clustering of seven T-phage species at three MOI levels (0.01, 0.1, 1.0; *n* = 3 per species per MOI). (**a**) PCA plot; colors indicate clusters, shapes indicate species. (**b**–**e**) LOSOCV performance comparing GC7 with established methods: (**b**) ARI, (**c**) NMI, (**d**) sampling score, (**e**) cluster count. Error bars indicate standard deviation.

To evaluate robustness under varying infection conditions, we combined data from three MOI levels (0.01, 0.1, and 1.0). This increased complexity, visualized in the PCA plot (Figure 3a, raw data: Table S5), reduced classification performance for all methods (Table S2). Using K-means, GC7 (ARI = 0.433 ± 0.055) nonetheless maintained substantially higher accuracy than VI, CI, or NMDS (ARI < 0.32) (Figure 2b–e). Notably, GC7 identified 13.9 ± 1.1 clusters (approximately two per species) while maintaining a very high sampling score (0.937 ± 0.035). In contrast, while established features produced cluster counts close to the true number of species, their very low sampling scores (<0.65) and ARIs indicate that these clusters were poorly formed, incorrectly mixing distinct species. Under these more complex conditions, GMM (ARI = 0.528 ± 0.088) outperformed K-means when applied to GC7 (Figure S5; Table S2). GMM identified 9.6 ± 1.7 clusters, suggesting its probabilistic approach is better suited to modeling the continuous phenotypic variation induced by the MOI gradient. DBSCAN and hierarchical clustering showed relatively low accuracy, consistent with other conditions.

### 3.4. Validation of Classification Framework Using Phages Extracted from Plaques

**Figure 4:**
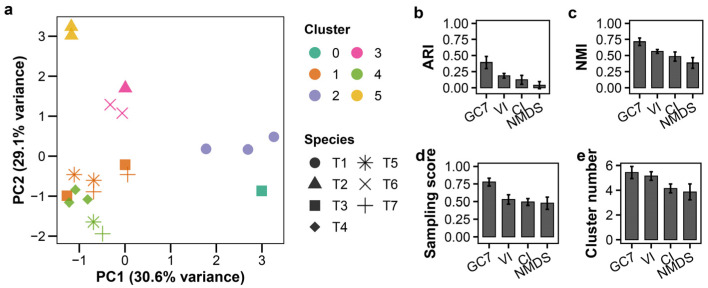
Growth curve-based classification of plaque-derived T-phages. K-means clustering of plaque-isolated T-phages (T1–T7; *n* = 3 per species except T6 with *n* = 2) without prior titer determination. (**a**) PCA plot; colors indicate clusters, shapes indicate species. (**b**–**e**) LOSOCV performance comparing GC7 with established methods: (**b**) ARI, (**c**) NMI, (**d**) sampling score, (**e**) cluster count. Error bars indicate standard deviation.

To approximate practical workflows, we validated the framework using phages propagated from individual plaques (n = 20 samples after excluding one T6 isolate). As expected, combining data from different MOIs introduced greater heterogeneity. As a result, the clustering performance was moderate and lay between the outcomes observed in experiments conducted as a single MOI and those performed at multiple MOIs (Figure 4a, raw data: Table S6). K-means clustering with GC7 yielded an ARI of 0.391 ± 0.094 (Figure 4b; Table S3). Strikingly, GMM produced an identical result (ARI = 0.391 ± 0.094), suggesting a robust clustering structure in the feature space (Figure S6). Both K-means and GMM with GC7 maintained superior accuracy and sampling scores compared to VI, CI, and NMDS (Figure 4b–d; Table S3). Other clustering methods performed poorly, consistent with previous results (Table S3). These results confirm GC7’s utility even when samples originate from less-standardized plaque-based preparations.

### 3.5. Classification of Phages Isolated from Sewage

**Figure 5:**
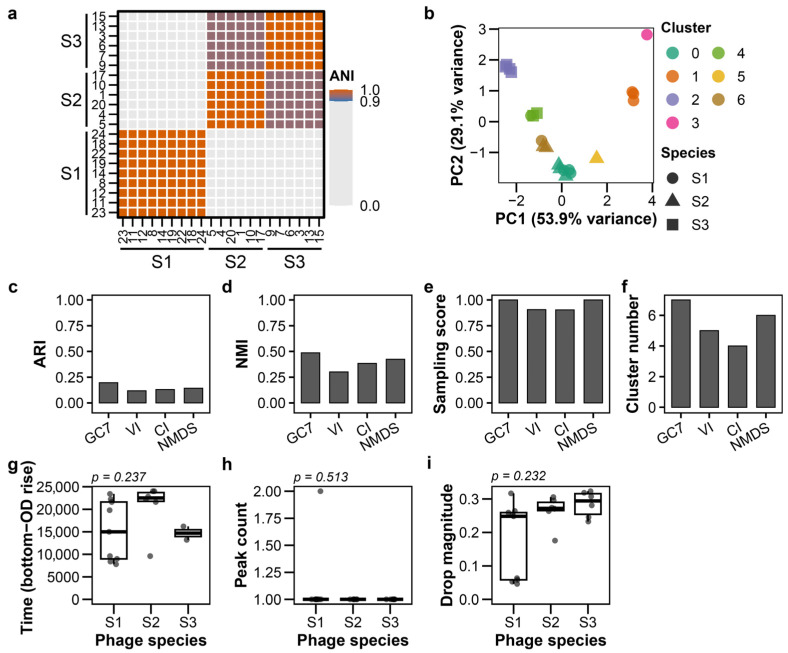
Growth curve-based classification of sewage-isolated phages. Analysis of 21 environmental phages isolated from sewage. (**a**) Average Nucleotide Identity (ANI) heatmap showing three genomic species clusters (ANI > 95% within clusters). (**b**) PCA plot of GC7 features; colors indicate clusters, shapes indicate ANI-defined species. (**c**–**f**) K-means clustering performance: (**c**) ARI, (**d**) NMI, (**e**) sampling score, (**f**) cluster count. (**g**–**i**) Feature distributions across species clusters: (**g**) time from bottom to OD rise, (**h**) peak count, (**i**) drop magnitude. *p* values from Kruskal–Wallis tests corrected by Holm are displayed above each panel.

To assess the applicability of the classification framework in practical settings, we applied it to 21 environmental phages isolated from sewage. The sewage-isolated phages exhibited similar plaque morphologies, making visual discrimination challenging and highlighting the need for alternative classification approaches (Figure S7; raw images: Supplementary Data). These phages were independently characterized by calculating their genomic Average Nucleotide Identity (ANI), which revealed three distinct species groups (Figure 5a, raw data: Table S7). Under these conditions, GC7 achieved the highest clustering accuracy among all tested feature sets, with an ARI of 0.196 (Figure 5b–d; Table S4). GMM clustering with GC7 produced results identical to K-means. Although the ARI values were limited, GC7 (using K-means or GMM) achieved a perfect sampling score (1.0), successfully identifying all three species. In contrast, VI and CI did not achieve perfect sampling scores (Figure 5e). Both DBSCAN and hierarchical clustering consistently showed lower performance (Figure S8). The absence of species-specific patterns in the distributions of time from bottom to OD rise, peak count, and drop magnitude (*p* > 0.05) may have contributed to the limited clustering accuracy (ARI = 0.196) observed for phages isolated from sewage (Figure 5g–i; Figure S9).

## 4. Discussion

This study demonstrates that bacterial growth curve-based classification using seven biologically meaningful features (GC7) enables effective discrimination of bacteriophage species. This result validates the utility of our approach as an upstream screening tool in phage isolation workflows. With T-phages at MOI 0.01, K-means clustering with GC7 outperformed established metrics, including the Virulence Index (VI), Centroid Index (CI), and NMDS-derived coordinates (ARI = 0.881 ± 0.057 vs. <0.6 for other methods). Importantly, when applied to sewage-isolated phages, our method achieved complete species detection (sampling score = 1.0), successfully identifying all three genomically distinct species despite limited overall clustering accuracy (ARI = 0.196). These results support the utility of GC7-based classification for the rapid triage of environmental phage samples prior to resource-intensive downstream characterization.

The superior performance of GC7 stems from its multi-dimensional, biologically informed design, which captures complementary aspects of phage–host interactions. The seven features exhibited low to moderate pairwise correlations (all r < 0.8), indicating that each metric provides distinct information across three critical phases. Lysis kinetics reflect infection synchrony and burst dynamics, lysis efficiency indicates phage productivity, and post-lysis dynamics reveal patterns of resistant mutant emergence and phage–bacteria coevolution. This design preserves phase-specific biological information that is unavoidably lost when growth curve data are compressed to single scalar values (VI, CI) or low-dimensional projections (NMDS). While VI and CI effectively summarize overall virulence for post-isolation characterization [19,20], they inherently discard the temporal information required to distinguish multiple unknown phages during initial isolation. The structured, biologically informed dimensionality of GC7 enables the more nuanced discrimination required for upstream screening, where multiple unknown phages must be rapidly triaged before detailed analyses are performed.

As expected, classification performance decreased under more complex conditions. When multiple MOI levels were combined, the ARI dropped substantially (from 0.881 to 0.433). This reflects the biological reality that identical phages produce different growth curves at varying MOIs due to differences in infection synchrony and dynamics. However, sampling scores remained high (>0.77), indicating successful detection of all species present. This distinction is critical for upstream screening. The goal at this preliminary stage is to identify which distinct phage types require further investigation, not necessarily to achieve perfect classification. For plaque-derived phages, where precise titering is unavailable and sample preparation varies, performance was intermediate (ARI = 0.391), yet GC7 maintained superior accuracy compared to established features. For sewage isolates, while overall clustering accuracy was limited (ARI = 0.196), the perfect sampling score (1.0) demonstrates a significant practical advantage. The low ARI combined with the perfect sampling score indicates over-clustering: samples from the same species were split across multiple clusters, but each species was represented in at least one distinct cluster. Consequently, all three species could be recovered by selecting one representative from each cluster, resulting in the perfect sampling score. This indicates that GC7-based classification excels at identifying species diversity, which is the primary goal of upstream screening, even when precise cluster assignments are challenging due to heterogeneous environmental conditions. The goal at this stage is to identify all distinct species present, not to achieve perfect cluster homogeneity. The analysis yielded 7 clusters from 21 samples, implying that detailed characterization of 7 representative isolates (33% of total) would be sufficient to capture all species diversity, thereby avoiding redundant analysis of the remaining 14 samples. During environmental isolation, phage concentrations are unknown and variable. GC7-based classification can flag sample diversity, enabling strategic prioritization for genomic sequencing while avoiding redundant analysis of closely related isolates.

Among the four clustering algorithms tested, K-means and GMM consistently outperformed DBSCAN and hierarchical methods. Under well-controlled conditions (single MOI, standardized titers), both achieved high accuracy (ARI > 0.75). GMM demonstrated superior performance under complex conditions (ARI = 0.528 vs. 0.433 for K-means with multiple MOIs), likely reflecting its probabilistic framework, which models continuous phenotypic variation more flexibly than K-means’ hard clustering. The convergence of K-means and GMM results for plaque-derived and sewage phages (identical ARIs) suggests a robust clustering structure within the GC7 feature space. For practical implementation, we recommend K-means or GMM.

The clinical relevance of this approach addresses an important bottleneck in personalized phage therapy. When suitable phages are unavailable in existing banks, de novo isolation from environmental sources is necessary [13,14]. Traditional plaque-based isolation followed by transmission electron microscopy, host range testing, and genomic sequencing requires weeks [15,16]. This timeline is incompatible with treating severe, antibiotic-resistant infections [2,3]. Our method enabled early-stage triage within 24 h for *E. coli* and T phages using standard microplate readers, allowing resources to be focused on diverse candidates before comprehensive characterization. This could accelerate the timeline from environmental sampling to therapeutic phage identification, facilitating both emergency treatments and the systematic expansion of phage bank collections.

Several limitations should be acknowledged. First, this validation was restricted to *E. coli* strains and their phages. Performance with clinically critical pathogens such as *P. aeruginosa*, *S. aureus*, and *K. pneumoniae* remains unknown, as host-dependent factors (e.g., growth kinetics, phage-resistant mutant frequency, metabolic state) may substantially influence growth curve patterns. Second, our largest test comprised seven T-phage species in controlled experiments; performance when environmental samples contain dozens of co-existing phages at varying titers is unclear. Third, the ground truth for sewage phages relied on genomic ANI clustering, which involves subjective threshold choices and may not perfectly correspond to functional or ecological species boundaries. Finally, our approach requires liquid culture-based monitoring, which may not capture phage behaviors apparent only on solid media or in biofilm-associated infections. Furthermore, phages that produce minimal or no lysis under these conditions, such as the excluded Samples 16 and 21, represent a detection limit for this method.

Regarding the scalability of GC7 to other bacterial species, the seven features capture fundamental aspects of phage-mediated lysis that are likely relevant across diverse bacterial hosts. For fast-growing pathogens with similar growth kinetics to *E. coli*, such as *K. pneumoniae*, *P. aeruginosa*, and *S. aureus* (all with doubling times of approximately 20–30 min under laboratory conditions), the current analytical framework should be directly applicable. However, host-dependent factors such as phage-resistant mutant frequency and lysis characteristics may require adjustment of detection thresholds, including the prominence threshold for peak detection and the OD change criteria for identifying lysis completion and regrowth onset. For slow-growing bacteria such as *Mycobacterium* species, the observation period would need to be extended.

Future development should prioritize two key directions. First, systematic validation across clinically relevant bacterial pathogens (e.g., *P. aeruginosa*, *S. aureus*, *K. pneumoniae*) is essential to establish pathogen-specific feature thresholds and potentially optimize feature selection for different hosts. Second, integration with automated high-throughput platforms that combine robotic liquid handling, real-time growth monitoring, and computational analysis pipelines would enable the processing of hundreds of environmental isolates within days rather than weeks.

## 5. Conclusions

This study establishes that GC7 features derived from bacterial growth curves enable effective unsupervised phage screening, validating their utility as an upstream prioritization tool. By quantifying lysis kinetics, efficiency, and post-lysis dynamics, GC7 outperforms established approaches and maintains high species detection even under heterogeneous conditions mirroring real-world sampling. This method provides actionable triage information, complementing essential downstream analyses. As phage therapy advances toward systematic clinical implementation, efficient discovery becomes paramount. Our approach enables the rapid identification of distinct candidates worthy of detailed characterization, reducing redundant analysis and potentially accelerating the turnaround time for personalized therapy. This capacity is critical for treating severe multidrug-resistant infections. Future validation across diverse pathogens and integration with automated platforms could transform this proof-of-concept into a comprehensive phage discovery pipeline, ultimately contributing to sustainable phage therapeutic development in the post-antibiotic era.

## Data Availability

Sequencing data are available in the NCBI SRA under accession number PRJNA1356948. All other data are provided in the main text and Supplementary Materials.

## Supplementary Materials

The following supporting information can be downloaded at: https://www.mdpi.com/article/10.3390/v18010092/s1, Figure S1: Schematic representation of GC7 features and sensitivity analysis for OD bottom detection threshold; Figure S2: Plaque morphology and taxonomic relationships of T-phages; Figure S3: Feature distributions and NMDS analysis for T-phages at multiplicity of infection (MOI) 0.01; Figure S4: Performance metrics of GC7-based clustering at MOI 0.01 using four algorithms; Figure S5: Performance metrics of GC7-based clustering across multiple MOI conditions (0.01, 0.1, and 1); Figure S6: Performance metrics of GC7-based clustering for plaque-derived phages; Figure S7: Plaque morphologies of sewage-isolated phages; Figure S8: Performance metrics of GC7-based clustering for sewage-isolated phages; Figure S9: Feature distributions for sewage-isolated phages; Table S1: Clustering performance metrics for T-phage samples at MOI 0.01; Table S2: Clustering performance metrics for T-phages across combined MOI conditions; Table S3: Clustering performance metrics for plaque-derived T-phage samples; Table S4: Clustering performance metrics for sewage-isolated phages; Table S5: Growth curve data of MOI controlled experiments; Table S6: Growth curve data of T-phages from plaques; Table S7: Growth curve data of phages from sewage; Supplementary Methods: Calculation of sampling scores; Supplementary Data: Raw images of phage plaques; Supplementary Code: Python and R scripts for growth curve analysis and clustering.

## Abbreviations

The following abbreviations are used in this manuscript:

AMR	Antimicrobial resistance
ANI	Average Nucleotide Identity
ARI	Adjusted Rand Index
BIC	Bayesian Information Criterion
CI	Centroid Index
DBSCAN	Density-based spatial clustering of applications with noise
GC7	Seven quantitative features extracted from growth curve
GMM	Gaussian Mixture Model
LB	Luria–Bertani
LOSOCV	Leave-One-Species-Out cross-validation
MOI	Multiplicity of infection
NBRC	Biological Resource Center, National Institute of Technology and Evaluation, Japan
NMDS	Non-metric multidimensional scaling
NMI	Normalized Mutual Information
OD_600_	Optical density at 600 nm
PERMANOVA	Permutational multivariate analysis of variance
PVDF	polyvinylidene difluoride
VI	Virulence Index
