# Supplementary material for: Feature-Based Growth Curve Classification Enables Efficient Phage Discrimination
Source: Viruses. 2026 Jan 9;18(1):92. doi: 10.3390/v18010092 (PMC12846622; doi:10.3390/v18010092)
Supplement: Supplementary file 1 [file viruses-18-00092-s001.zip › supplementary_materials_revision.pdf]

Supplementary Materials

Feature-Based Growth Curve Classification Enables Efficient Phage Discrimination

Yuma Oka[1,*], Keidai Miyakawa[1], Moe Yamazaki[1], Yuki Maruyama[1]

[1]H.U. Group Research Institute G.K., Fuchigami 50, Akiruno, Tokyo, Japan

*Corresponding author

E-mail: yuma.oka@hugp.com

Supplementary Figures and Tables

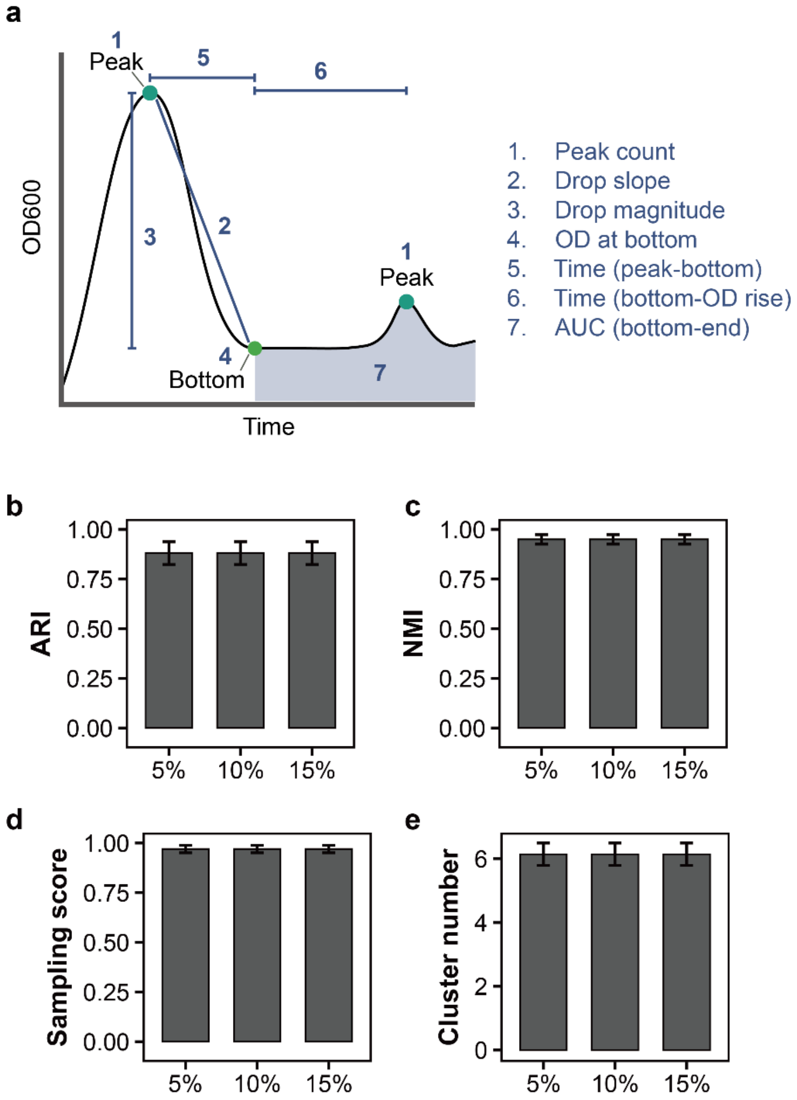

Figure S1. Schematic representation of GC7 features and sensitivity analysis for OD bottom detection threshold. (a) Schematic diagram of a bacterial growth curve illustrating the seven GC7 features. (b-e) Sensitivity analysis of the lysis rate threshold for defining OD at bottom. The default threshold (10% of maximum lysis rate) was compared with alternative

thresholds (5% and 15%). Leave-One-Species-Out cross-validation was performed using GC7 features with K-means clustering on MOI 0.01 data: (b) Adjusted Rand Index (ARI), (c) Normalized Mutual Information (NMI), (d) sampling score, (e) cluster number.

a

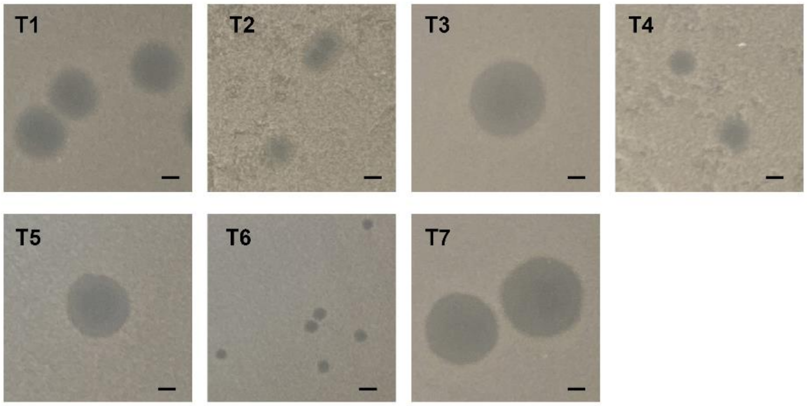

b

Phage	Class	Order	Family	Subfamily	Genus	Species
**T1**	*Caudoviricetes*	Not assigned	*Drexlerviridae*	*Tunavirinae*	*Tunavirus*	*Tunavirus T1*
**T2**	*Caudoviricetes*	*Pantevenvirales*	*Straboviridae*	*Tevenvirinae*	*Tequatrovirus*	*Tequatrovirus T2*
**T3**	*Caudoviricetes*	*Autographivirales*	*Autographiviridae*	*Studiervirinae*	*Teetrevirus*	*Teetrevirus T3*
**T4**	*Caudoviricetes*	*Pantevenvirales*	*Straboviridae*	*Tevenvirinae*	*Tequatrovirus*	*Tequatrovirus T4*
**T5**	*Caudoviricetes*	Not assigned	*Demerecviridae*	*Demerecviridae*	*Tequintavirus*	*Tequintavirus T5*
**T6**	*Caudoviricetes*	*Pantevenvirales*	*Straboviridae*	*Tevenvirinae*	*Tequatrovirus*	*Tequatrovirus T6*
**T7**	*Caudoviricetes*	*Autographivirales*	*Autotranscriptaviridae*	*Studiervirinae*	*Teseptimavirus*	*Teseptimavirus T7*

c

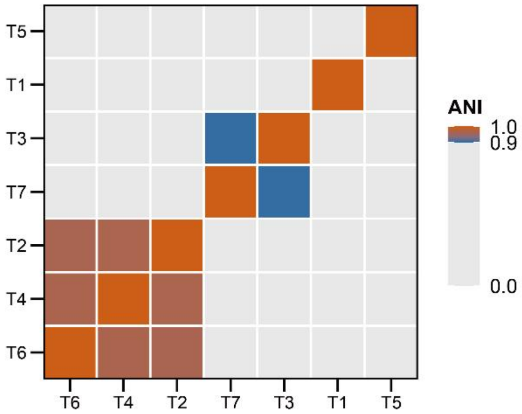

Figure S2. Plaque morphology and taxonomic relationships of T-phages

(a) Plaque morphologies of T-phages (T1–T7). Scale bar: 1mm (b) Taxonomic classification of T-phages. (c) Average Nucleotide Identity (ANI) heatmap showing genomic relationships among T-phages.

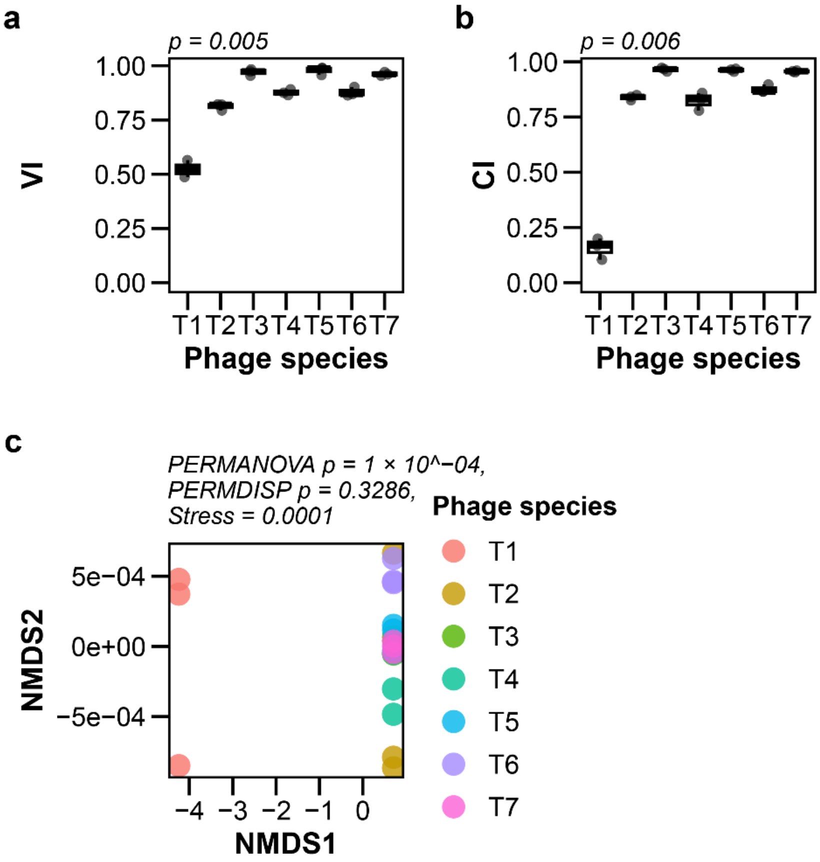

Figure S3. Feature distributions and NMDS analysis for T-phages at multiplicity of infection (MOI) 0.01

(a) Virulence Index (VI) and (b) Centroid Index (CI) distributions among T-phage species. (c) Two-dimensional non-metric multidimensional scaling (NMDS) ordination plot. P values from PERMANOVA (testing for differences among species) and PERMDISP (testing for homogeneity of dispersion) along with stress value are shown above the panel.

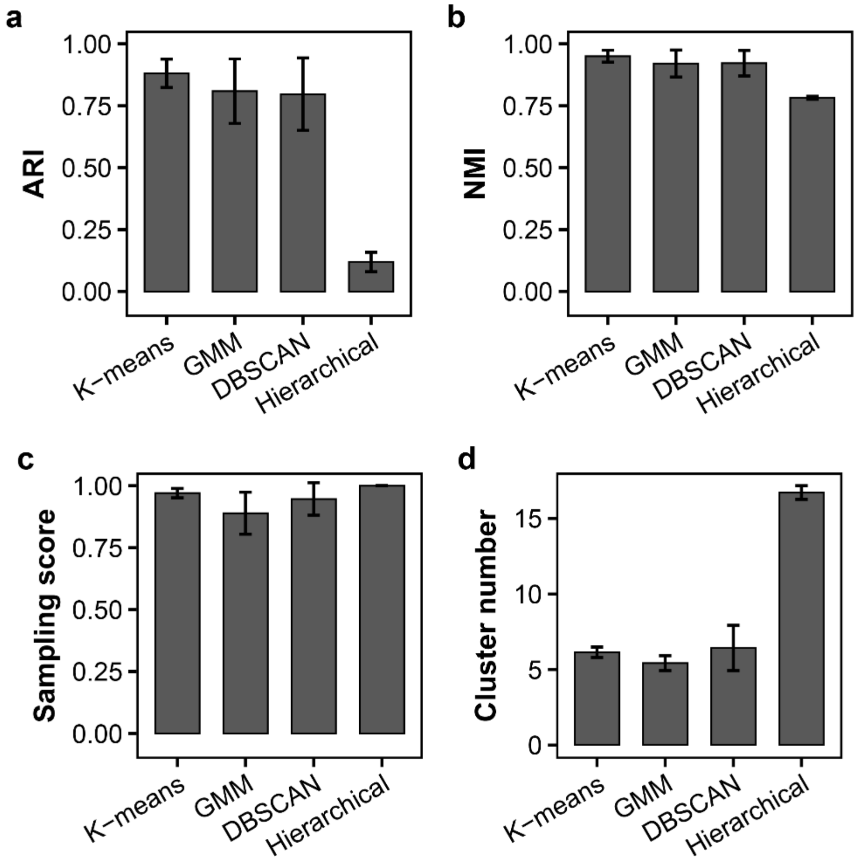

Figure S4. Performance metrics of GC7-based clustering at MOI 0.01 using four algorithms

Performance of K-means, Gaussian Mixture Model (GMM), Density-Based Spatial Clustering of Applications with Noise (DBSCAN), and Hierarchical clustering evaluated using: (a) ARI, (b) NMI, (c) Sampling score, (d) Number of clusters identified.

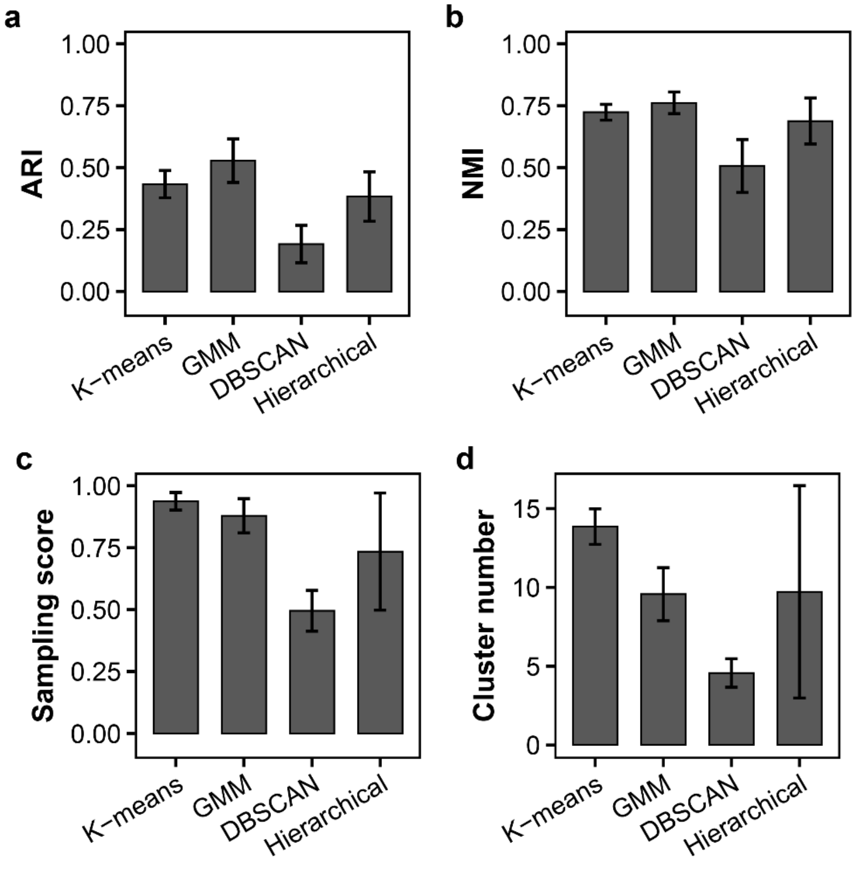

Figure S5. Performance metrics of GC7-based clustering across multiple MOI conditions (0.01, 0.1, and 1)

Performance of K-means, GMM, DBSCAN, and Hierarchical clustering evaluated using: (a) ARI, (b) NMI, (c) Sampling score, (d) Number of clusters identified.

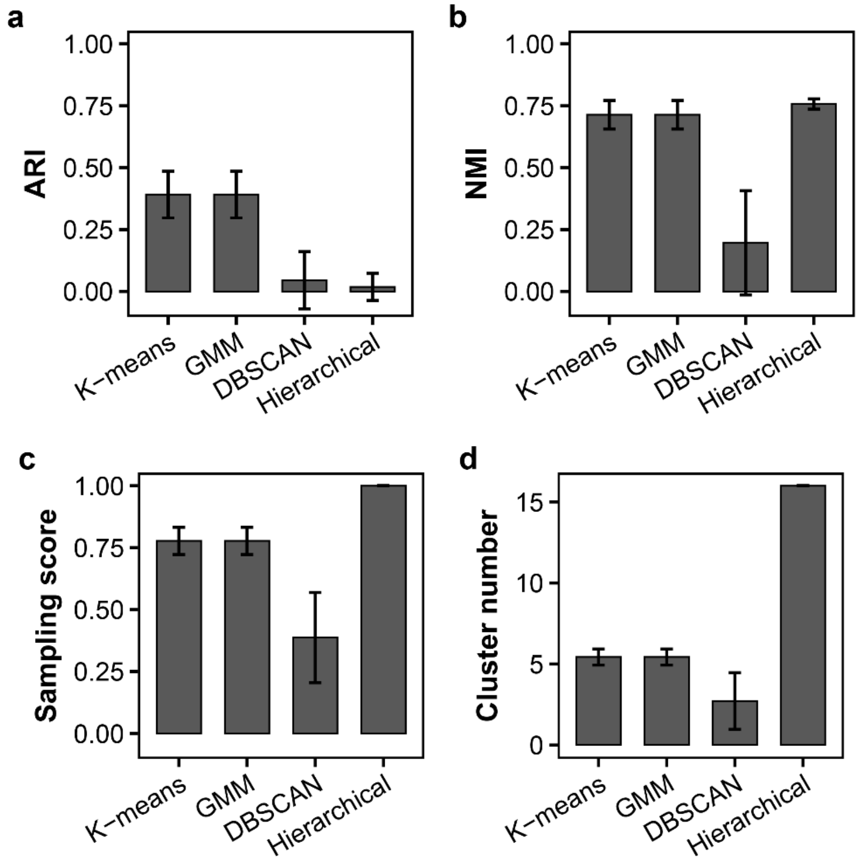

Figure S6. Performance metrics of GC7-based clustering for plaque-derived phages

Performance of K-means, GMM, DBSCAN, and Hierarchical clustering evaluated using: (a) ARI, (b) NMI, (c) Sampling score, (d) Number of clusters identified.

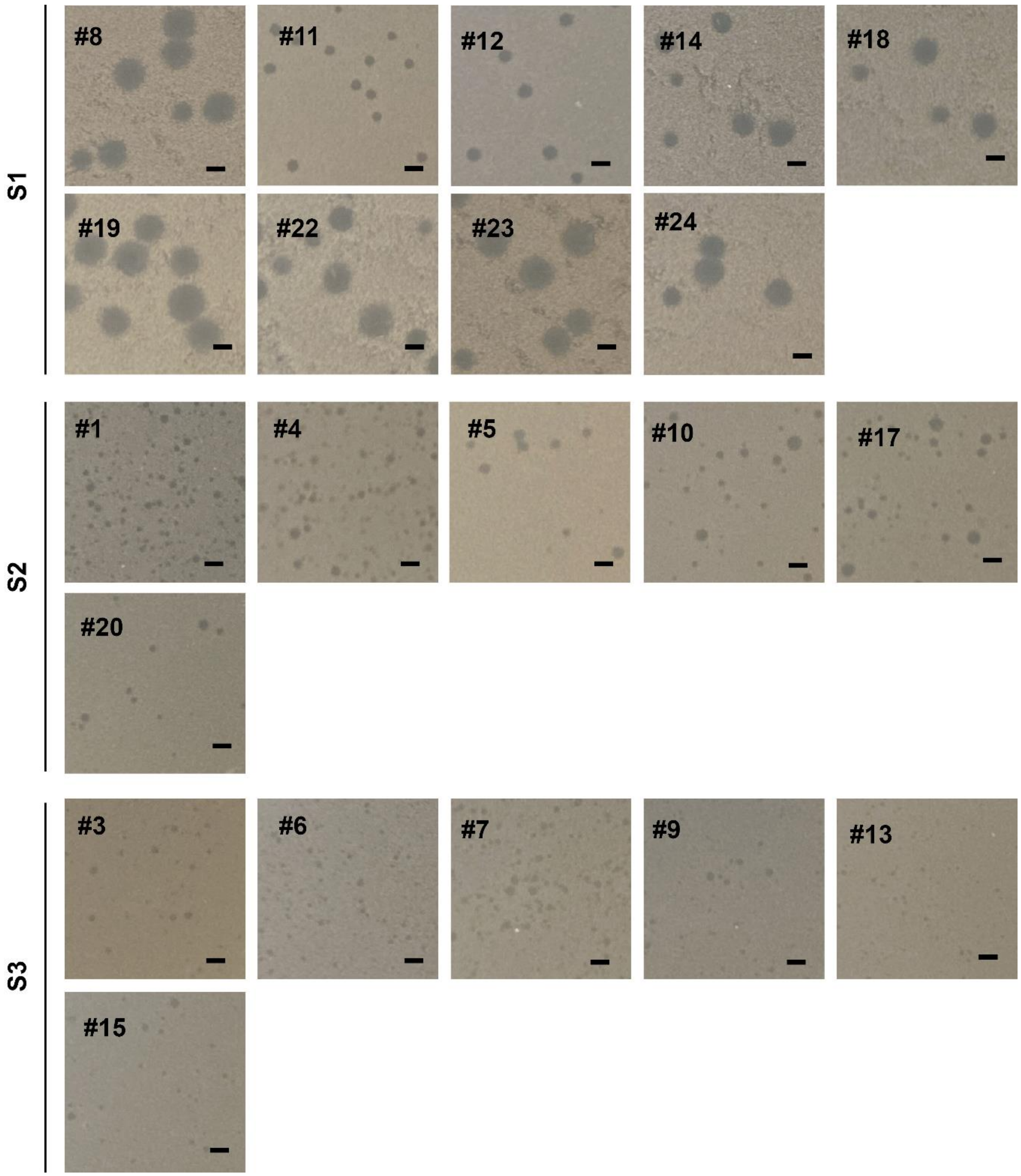

Figure S7. Plaque morphologies of sewage-isolated phages

Plaques are grouped by ANI-defined species (S1, S2, and S3). Scale bar: 1 mm.

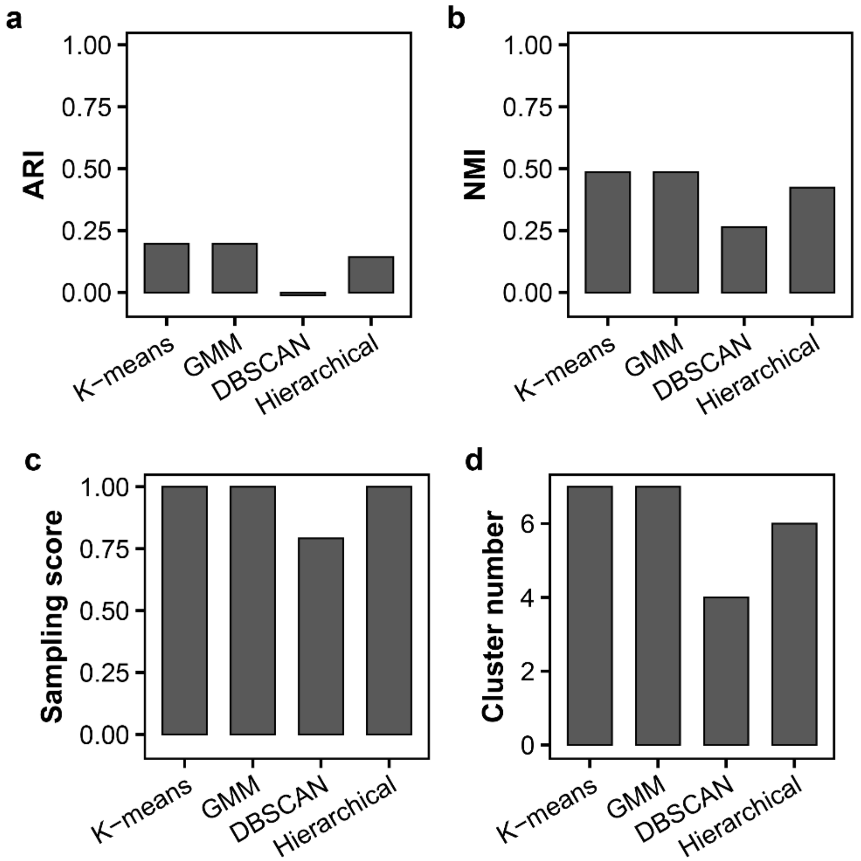

Figure S8. Performance metrics of GC7-based clustering for sewage-isolated phages

Performance of K-means, GMM, DBSCAN, and Hierarchical clustering evaluated using: (a) ARI, (b) NMI, (c) Sampling score, (d) Number of clusters identified.

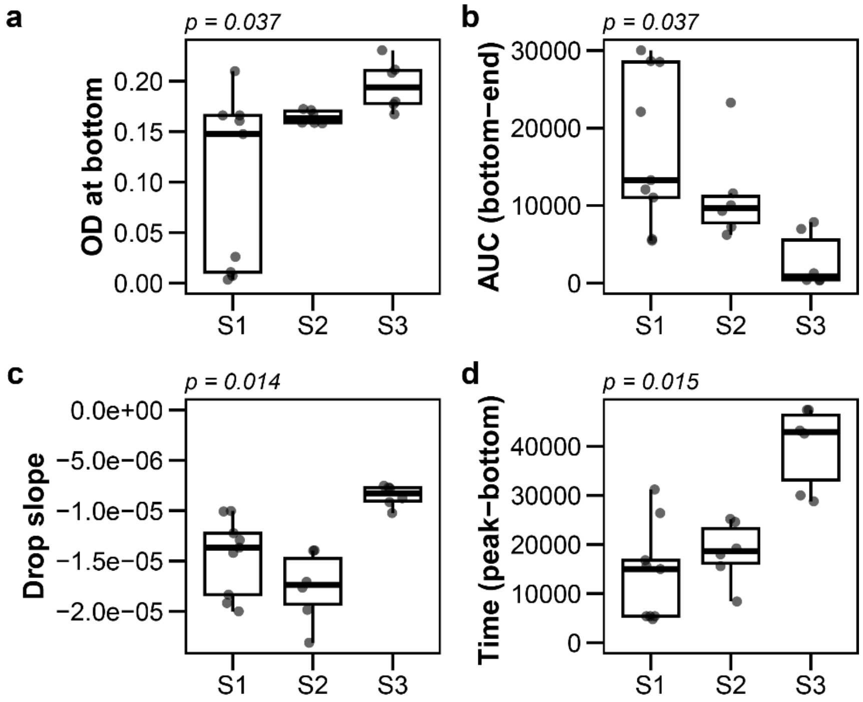

Figure S9. Feature distributions for sewage-isolated phages

Boxplots showing distributions of selected features: (a) OD at bottom, (b) Post-lysis AUC (from bottom to endpoint), (c) Drop slope, (d) Time from peak to bottom. Features that showed significant differences among species groups ($P < 0.05$ by Kruskal-Wallis test).

Table S1. Clustering performance metrics for T-phage samples at MOI 0.01

Four clustering algorithms were evaluated: K-means, Gaussian Mixture Model (GMM), Density-Based Spatial Clustering of Applications with Noise (DBSCAN), and Hierarchical clustering with Ward's linkage. Leave-One-Species-Out cross-validation was performed using GC7 (seven-feature set), Virulence Index (VI), Centroid Index (CI), and two-dimensional NMDS coordinates. Values represent mean (standard deviation) across seven cross-validation iterations (n = 6 species per iteration).

Algorithm	Feature	ARI (SD)	NMI (SD)	Sampling Score (SD)	Cluster Number (SD)
K-means	GC7	0.881 (0.057)	0.950 (0.024)	0.970 (0.019)	6.1 (0.3)
K-means	VI	0.491 (0.120)	0.790 (0.083)	0.648 (0.061)	4.0 (0.5)
K-means	CI	0.380 (0.081)	0.700 (0.048)	0.593 (0.059)	4.1 (0.6)
K-means	NMDS	0.395 (0.132)	0.714 (0.076)	0.714 (0.084)	5.1 (0.6)
GMM	GC7	0.809 (0.130)	0.920 (0.054)	0.889 (0.085)	5.4 (0.5)
GMM	VI	0.333 (0.103)	0.653 (0.117)	0.452 (0.075)	2.7 (0.5)
GMM	CI	0.363 (0.050)	0.694 (0.057)	0.490 (0.073)	3.1 (0.8)
GMM	NMDS	0.182 (0.151)	0.734 (0.056)	0.920 (0.104)	12.6 (3.4)
DBSCAN	GC7	0.797 (0.146)	0.922 (0.052)	0.946 (0.066)	6.4 (1.5)
DBSCAN	VI	0.430 (0.075)	0.757 (0.043)	0.595 (0.082)	5.0 (1.5)
DBSCAN	CI	0.272 (0.077)	0.634 (0.104)	0.610 (0.147)	5.0 (1.7)
DBSCAN	NMDS	0.421 (0.167)	0.779 (0.066)	0.667 (0.000)	6.4 (2.3)
Hierarchical	GC7	0.119 (0.039)	0.782 (0.006)	1.000 (0.000)	16.7 (0.5)
Hierarchical	VI	0.021 (0.053)	0.647 (0.155)	0.810 (0.301)	12.7 (6.8)
Hierarchical	CI	0.079 (0.037)	0.773 (0.012)	1.000 (0.000)	17.0 (0.0)
Hierarchical	NMDS	0.079 (0.037)	0.773 (0.012)	1.000 (0.000)	17.0 (0.0)

Table S2. Clustering performance metrics for T-phages across combined MOI conditions

Four clustering algorithms were evaluated: K-means, GMM, DBSCAN, and Hierarchical clustering with Ward's linkage. Leave-One-Species-Out cross-validation was performed using GC7, VI, CI, and NMDS coordinates across samples from MOI 0.01, 0.1, and 1. Values represent mean (standard deviation) across seven cross-validation iterations (n = 6 species per iteration).

Algorithm	Feature	ARI (SD)	NMI (SD)	Sampling Score (SD)	Cluster Number (SD)
K-means	GC7	0.433 (0.055)	0.724 (0.032)	0.937 (0.035)	13.9 (1.1)
K-means	VI	0.314 (0.059)	0.592 (0.064)	0.602 (0.014)	5.1 (0.3)
K-means	CI	0.317 (0.047)	0.597 (0.033)	0.605 (0.064)	5.9 (0.6)
K-means	NMDS	0.300 (0.035)	0.570 (0.020)	0.646 (0.034)	5.9 (0.8)
GMM	GC7	0.528 (0.088)	0.762 (0.044)	0.879 (0.069)	9.6 (1.7)
GMM	VI	0.372 (0.066)	0.619 (0.077)	0.468 (0.057)	2.9 (0.3)
GMM	CI	0.414 (0.043)	0.673 (0.026)	0.574 (0.055)	4.7 (0.9)
GMM	NMDS	0.333 (0.041)	0.585 (0.039)	0.576 (0.160)	4.9 (2.0)
DBSCAN	GC7	0.192 (0.075)	0.506 (0.107)	0.495 (0.082)	4.6 (0.9)
DBSCAN	VI	0.116 (0.113)	0.364 (0.180)	0.355 (0.103)	6.3 (2.2)
DBSCAN	CI	0.106 (0.031)	0.399 (0.077)	0.442 (0.072)	5.4 (1.3)
DBSCAN	NMDS	0.248 (0.116)	0.562 (0.157)	0.561 (0.120)	8.0 (2.3)
Hierarchical	GC7	0.383 (0.100)	0.688 (0.093)	0.734 (0.237)	9.7 (6.7)
Hierarchical	VI	0.147 (0.039)	0.413 (0.027)	0.350 (0.040)	2.1 (0.3)
Hierarchical	CI	0.182 (0.105)	0.449 (0.124)	0.435 (0.084)	2.9 (0.6)

Hierarchical	NMDS	0.162 (0.036)	0.482 (0.093)	0.546 (0.247)	9.6 (8.7)

Table S3. Clustering performance metrics for plaque-derived T-phage samples

Four clustering algorithms were evaluated: K-means, GMM, DBSCAN, and Hierarchical clustering with Ward's linkage. Leave-One-Species-Out cross-validation was performed using GC7, VI, CI, and NMDS coordinates across samples from MOI 0.01, 0.1, and 1. Values represent mean (standard deviation) across seven cross-validation iterations (n = 6 species per iteration).

Algorithm	Feature	ARI (SD)	NMI (SD)	Sampling Score (SD)	Cluster Number (SD)
K-means	GC7	0.391 (0.094)	0.714 (0.057)	0.777 (0.055)	5.4 (0.5)
K-means	VI	0.185 (0.037)	0.564 (0.030)	0.530 (0.067)	5.1 (0.3)
K-means	CI	0.122 (0.069)	0.483 (0.072)	0.494 (0.048)	4.1 (0.3)
K-means	NMDS	0.040 (0.054)	0.384 (0.083)	0.477 (0.086)	3.9 (0.6)
GMM	GC7	0.391 (0.094)	0.714 (0.057)	0.777 (0.055)	5.4 (0.5)
GMM	VI	0.124 (0.077)	0.405 (0.186)	0.355 (0.104)	2.9 (1.5)
GMM	CI	0.152 (0.053)	0.553 (0.066)	0.562 (0.072)	6.1 (1.1)
GMM	NMDS	0.059 (0.030)	0.482 (0.112)	0.531 (0.198)	5.9 (4.2)
DBSCAN	GC7	0.045 (0.115)	0.196 (0.210)	0.387 (0.182)	2.7 (1.7)
DBSCAN	VI	0.074 (0.035)	0.358 (0.101)	0.330 (0.007)	2.6 (0.5)
DBSCAN	CI	0.097 (0.040)	0.386 (0.158)	0.411 (0.111)	2.6 (0.7)
DBSCAN	NMDS	0.058 (0.025)	0.354 (0.146)	0.370 (0.106)	3.3 (1.2)
Hierarchical	GC7	0.019 (0.055)	0.757 (0.021)	1.000 (0.000)	16.0 (0.0)
Hierarchical	VI	0.029 (0.051)	0.599 (0.175)	0.714 (0.330)	10.3 (6.6)

Hierarchical	CI	0.040 (0.063)	0.624 (0.146)	0.775 (0.260)	10.4 (6.4)
Hierarchical	NMDS	0.033 (0.055)	0.762 (0.016)	1.000 (0.000)	16.0 (0.0)

Table S4. Clustering performance metrics for sewage-isolated phages

Four clustering algorithms were evaluated: K-means, GMM, DBSCAN, and Hierarchical clustering with Ward's linkage. Performance was assessed using GC7, VI, CI, and NMDS coordinates.

Algorithm	Feature	ARI	NMI	Sampling Score	Cluster Number
K-means	GC7	0.196	0.486	1	7
K-means	VI	0.118	0.301	0.905	5
K-means	CI	0.13	0.384	0.903	4
K-means	NMDS	0.143	0.423	1	6
GMM	GC7	0.196	0.486	1	7
GMM	VI	0.191	0.373	0.842	4
GMM	CI	0.13	0.384	0.903	4
GMM	NMDS	0.195	0.551	1	12
DBSCAN	GC7	-0.012	0.264	0.792	4
DBSCAN	VI	0.192	0.391	0.889	5
DBSCAN	CI	0.13	0.384	0.903	4
DBSCAN	NMDS	-0.017	0.2	0.677	3
Hierarchical	GC7	0.143	0.423	1	6
Hierarchical	VI	0.033	0.473	1	15
Hierarchical	CI	-0.017	0.184	0.556	2

Hierarchical	NMDS	0.139	0.527	1	14

Supplementary Methods

Calculation of sampling scores

Let $\mathcal{T}$ denote the set of sample types. For cluster k, let $n_{k,t}$ be the count of type $t \in \mathcal{T}$ in that cluster, and $N_k = \sum_{t \in \mathcal{T}} n_{k,t}$ the cluster size. The probability that a single random draw from cluster k yields type t is:

$$p_{k,t} = \frac{n_{k,t}}{N_k}.$$

Assuming one independent draw from each cluster, the probability that type t is observed at least once across all clusters is:

$$S_t = 1 - \prod_{k=1}^{K} \left(1 - p_{k,t}\right).$$

We summarize type-level coverage by the mean selection probability:

$$\bar{S} = \frac{1}{|\mathcal{T}|} \sum_{t \in \mathcal{T}} S_t.$$

A higher $\bar{S}$ indicates that the clustering is more representative of the underlying label distribution and better suited for downstream stratified sampling or phenotype grouping.
