# Supplementary figures and images for: Feature-Based Growth Curve Classification Enables Efficient Phage Discrimination
Source: Viruses. 2026 Jan 9;18(1):92. doi: 10.3390/v18010092 (PMC12846622; doi:10.3390/v18010092)

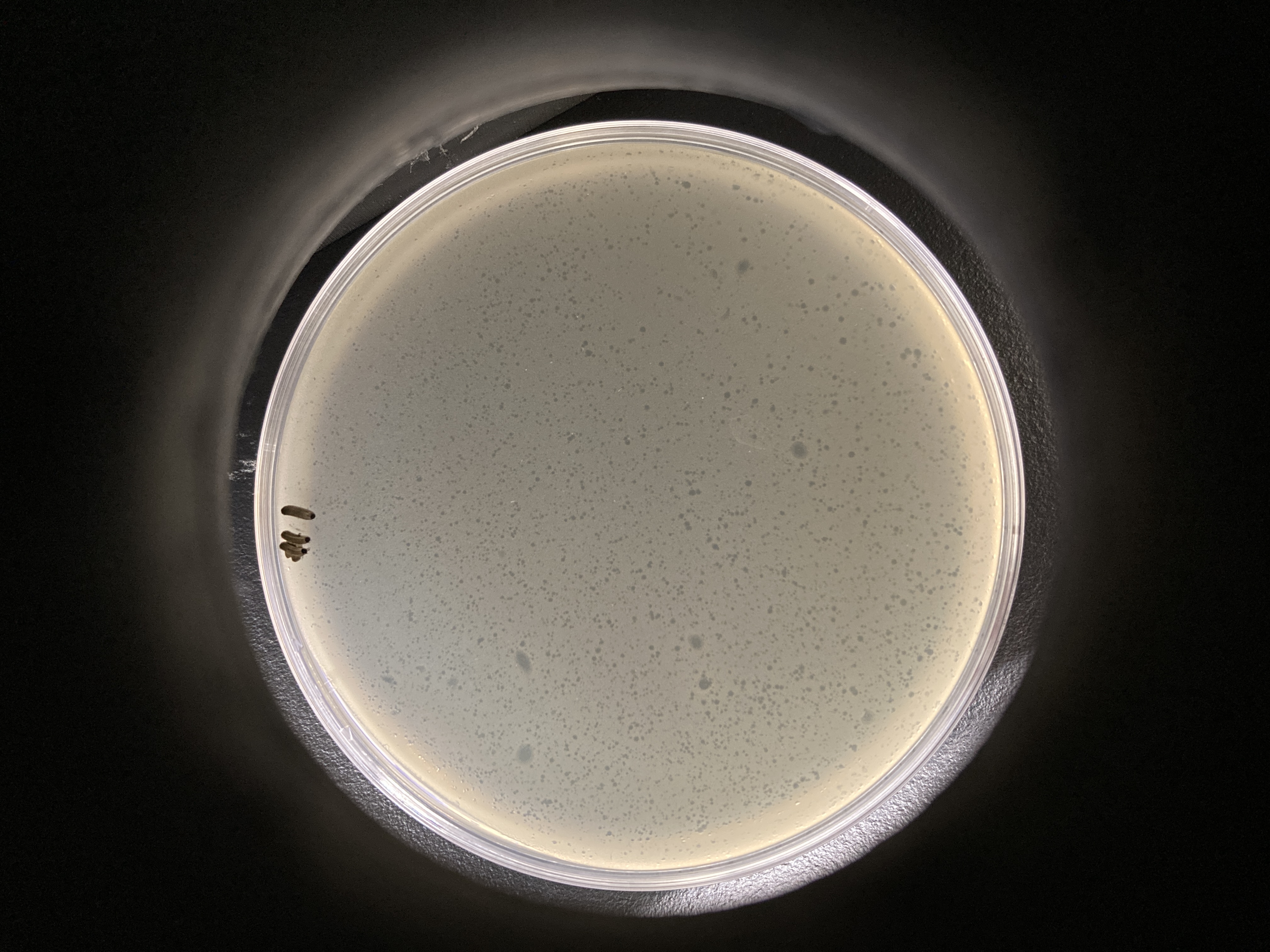

Supplement: Supplementary file 1 [file viruses-18-00092-s001.zip › No1.jpg]

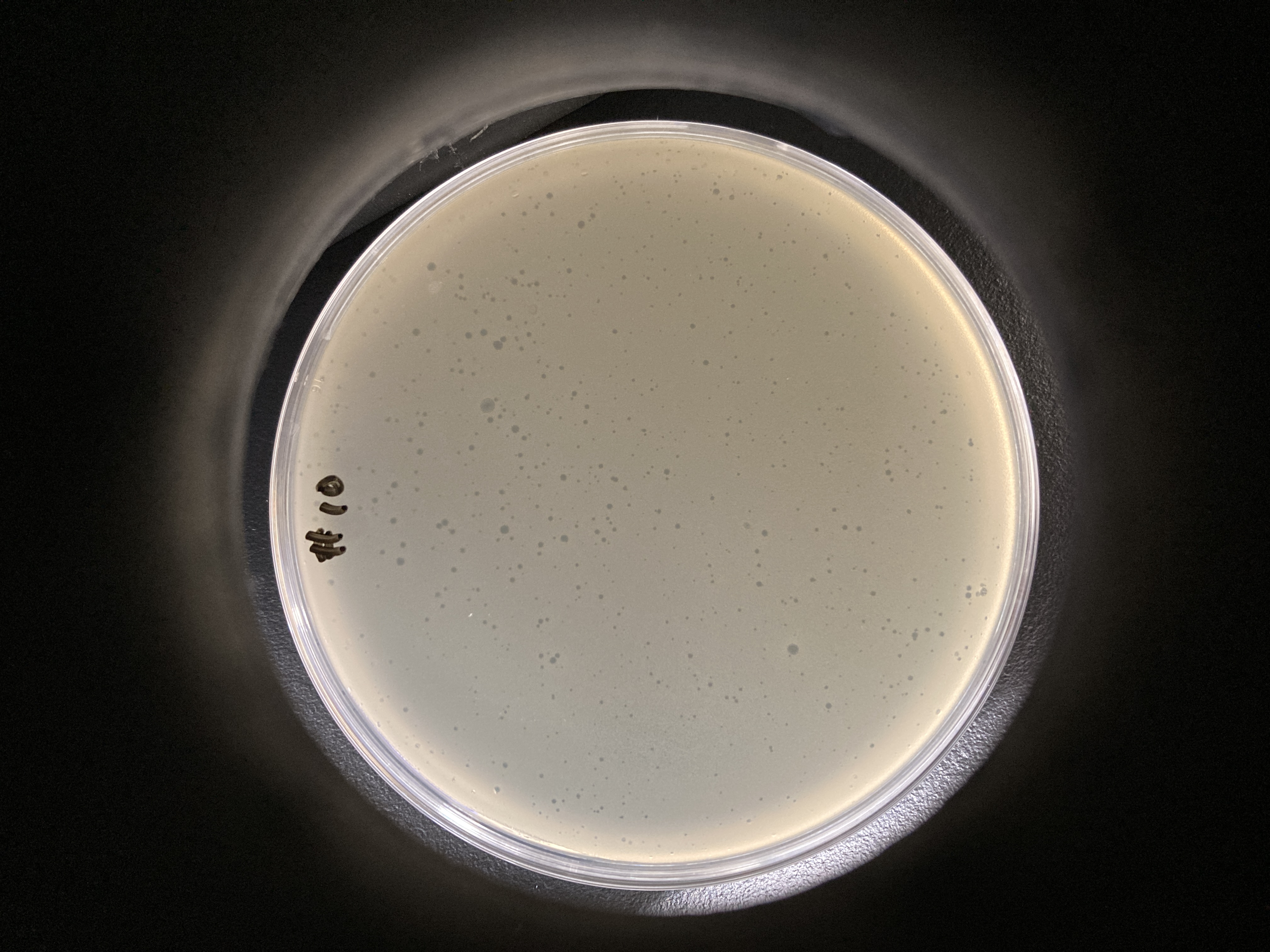

Supplement: Supplementary file 1 [file viruses-18-00092-s001.zip › No10.jpg]

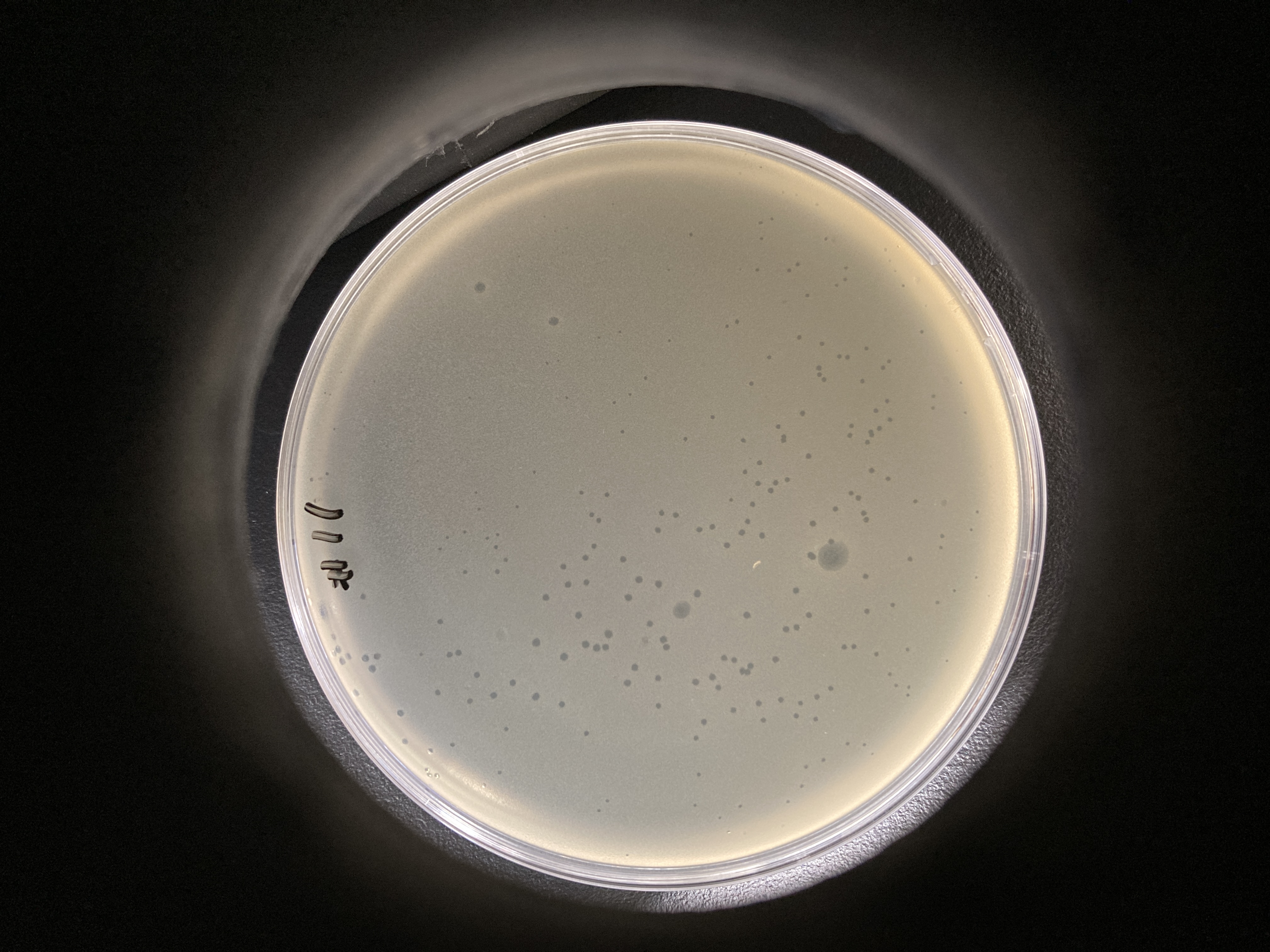

Supplement: Supplementary file 1 [file viruses-18-00092-s001.zip › No11.jpg]

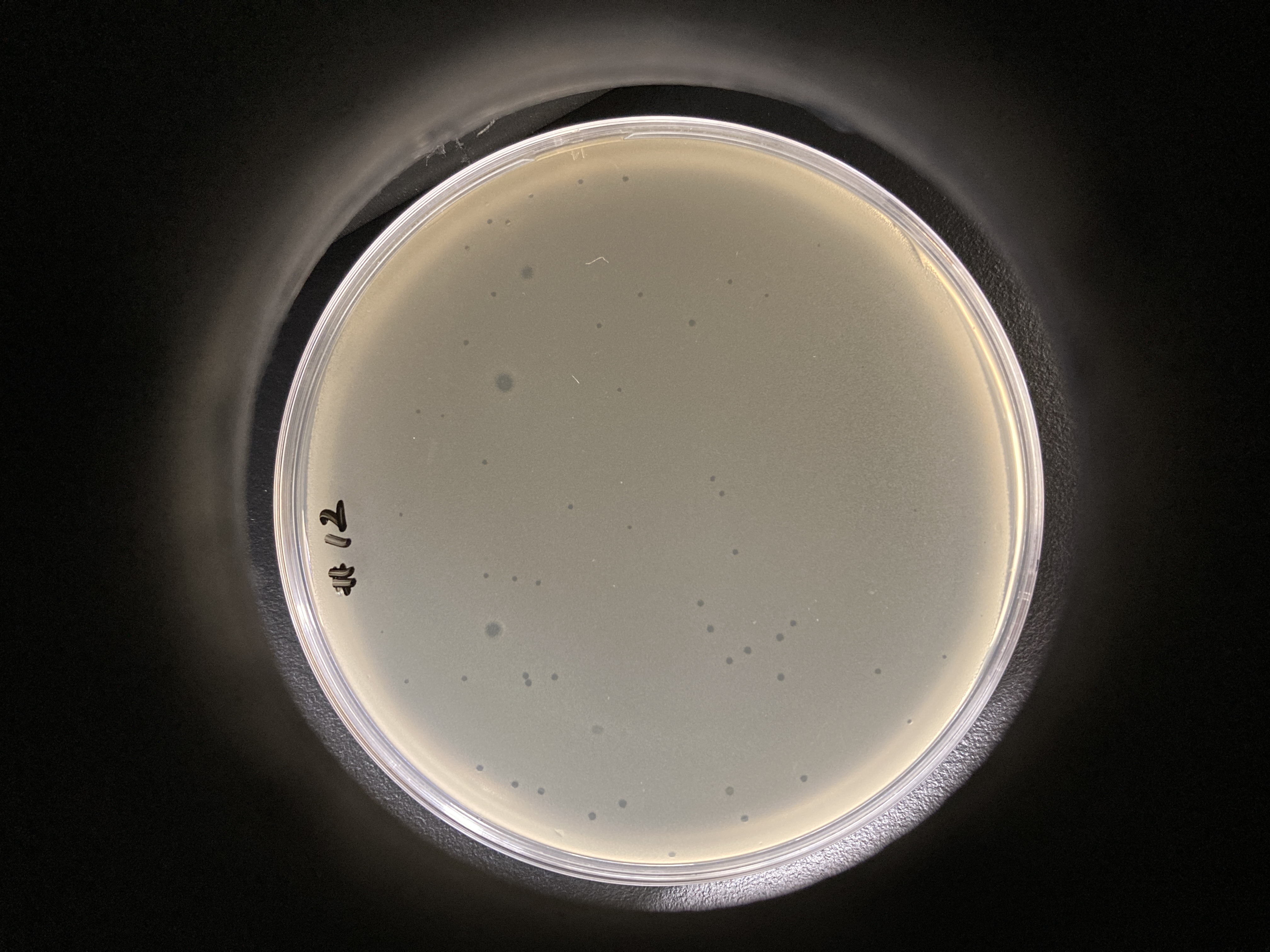

Supplement: Supplementary file 1 [file viruses-18-00092-s001.zip › No12.jpg]

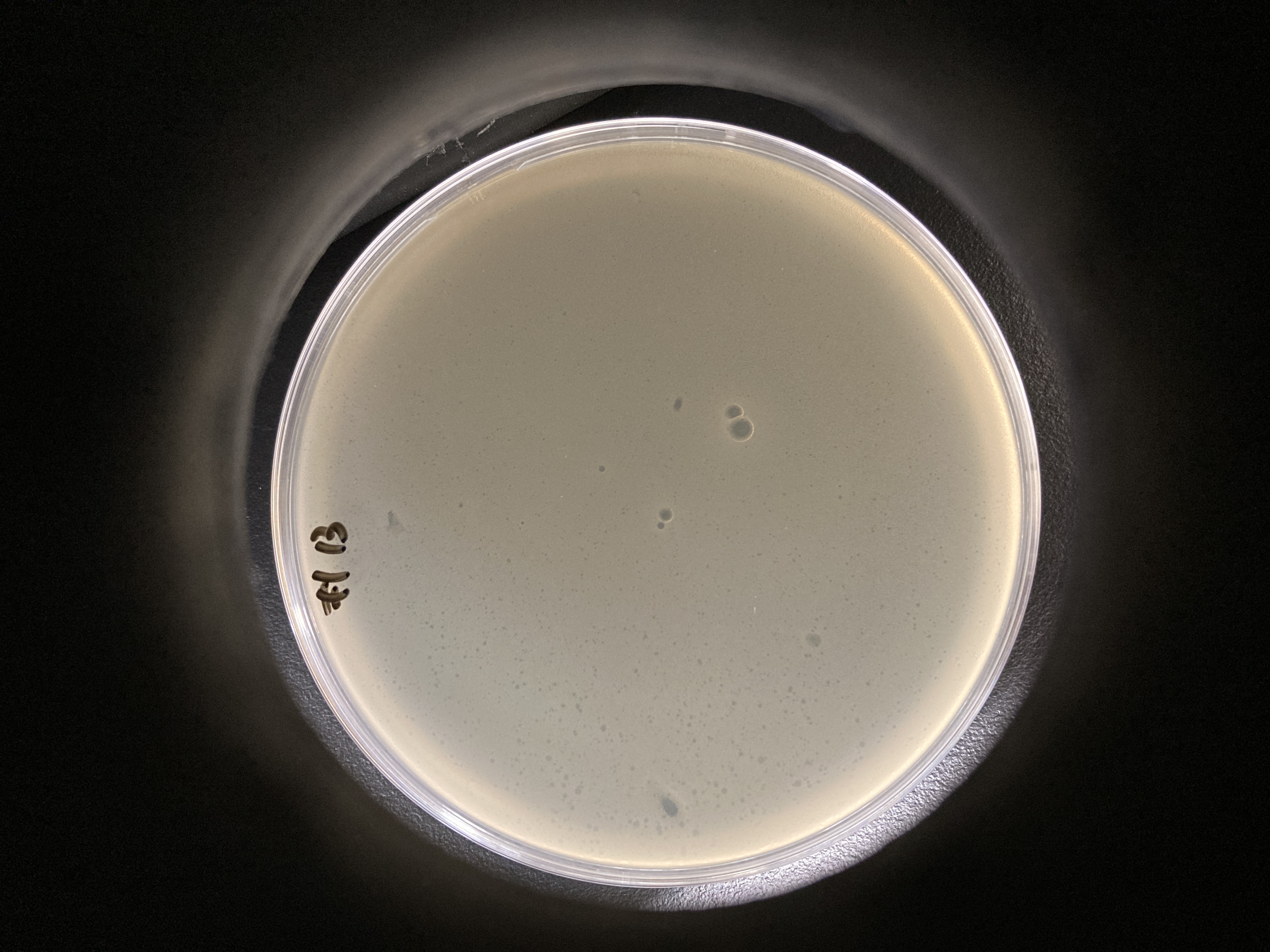

Supplement: Supplementary file 1 [file viruses-18-00092-s001.zip › No13.jpg]

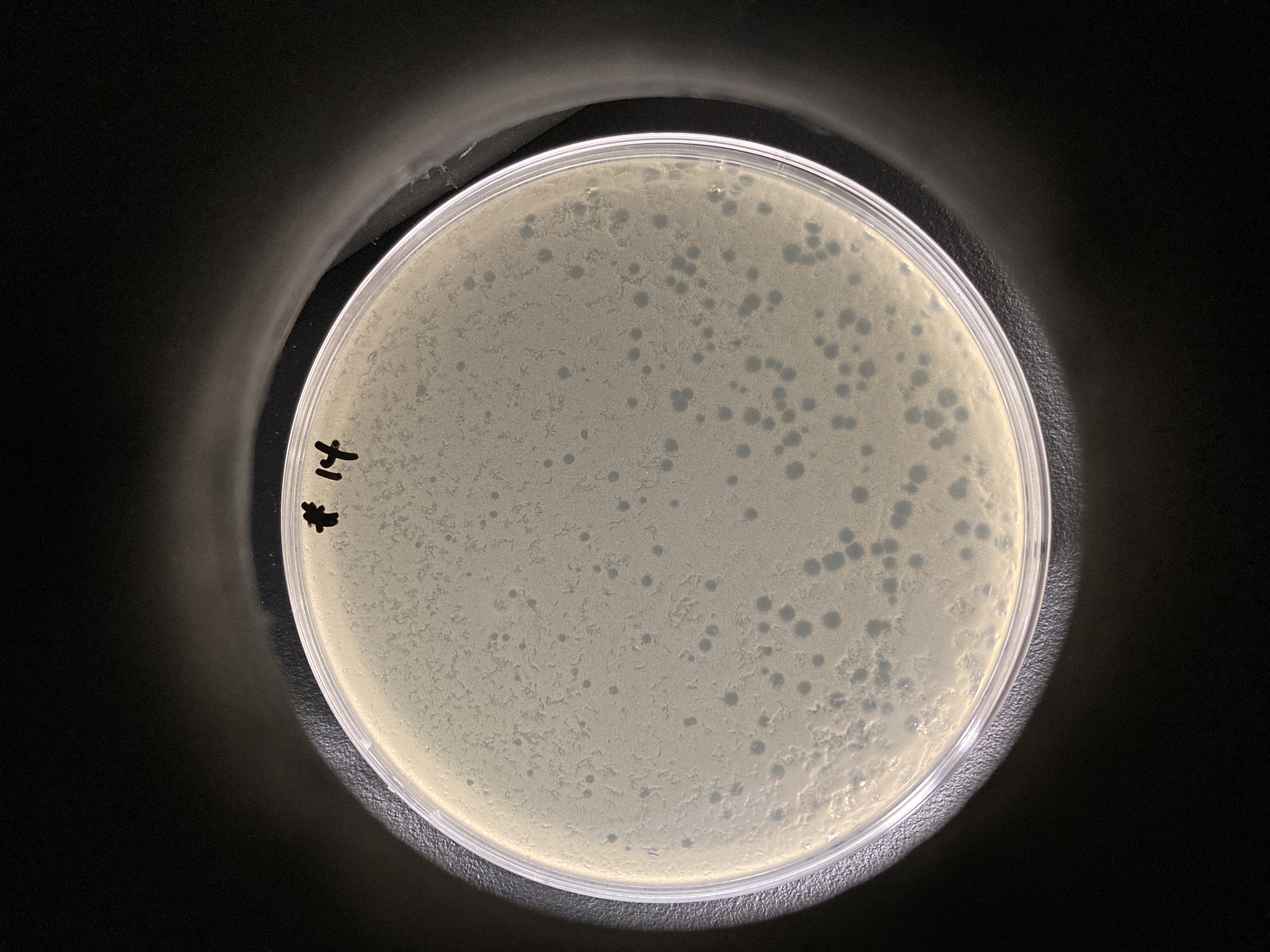

Supplement: Supplementary file 1 [file viruses-18-00092-s001.zip › No14.jpg]

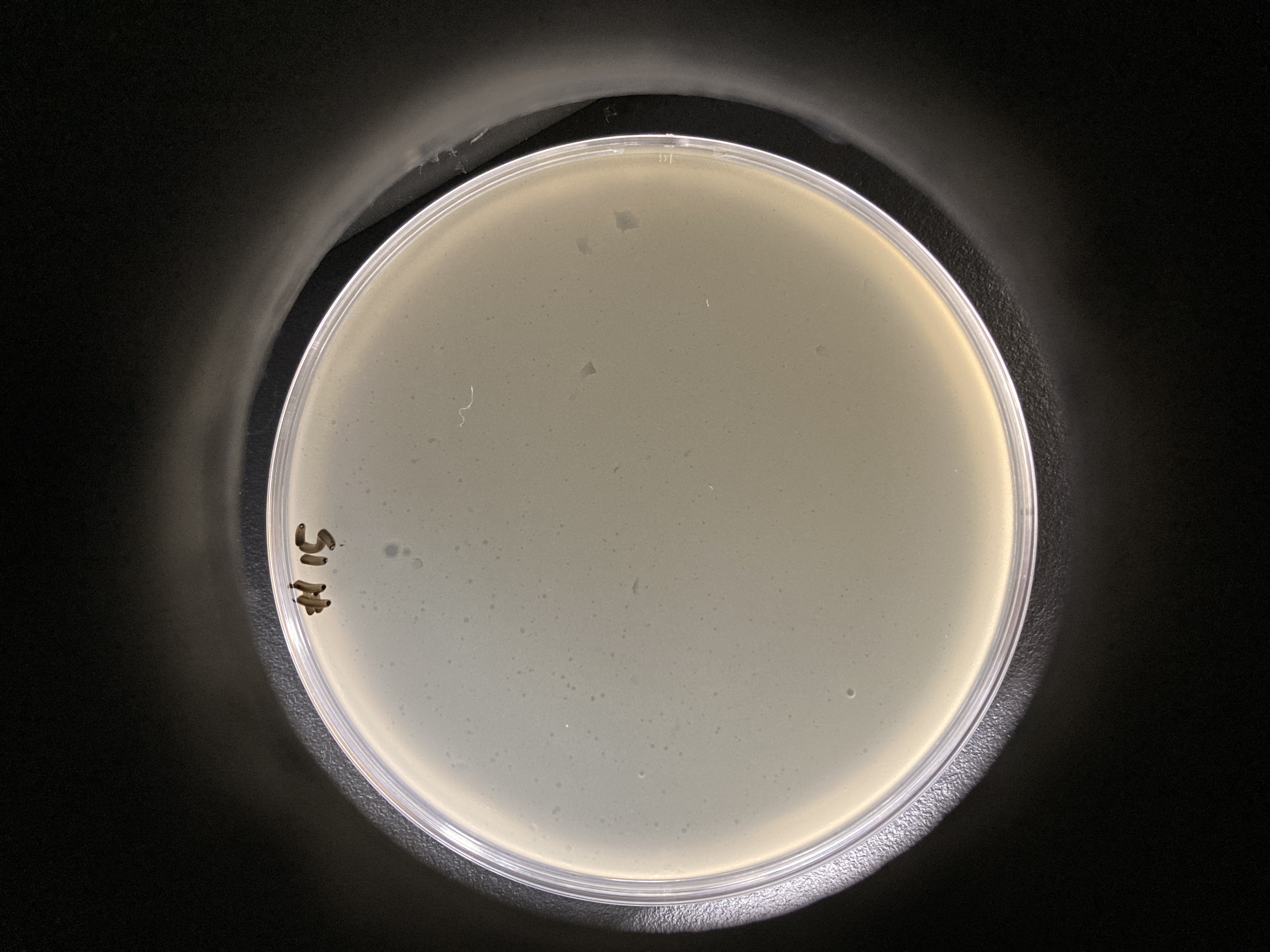

Supplement: Supplementary file 1 [file viruses-18-00092-s001.zip › No15.jpg]

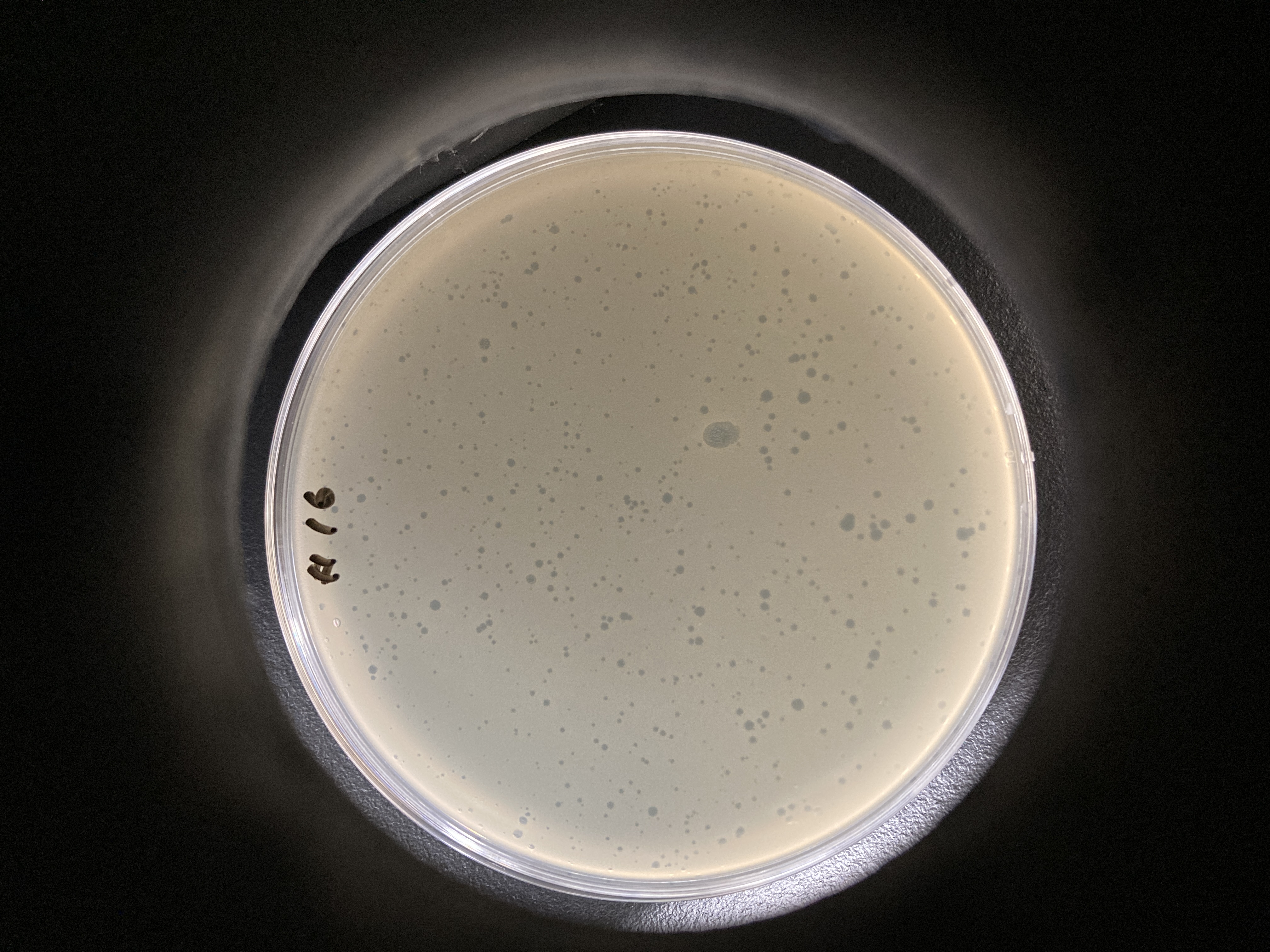

Supplement: Supplementary file 1 [file viruses-18-00092-s001.zip › No16.jpg]

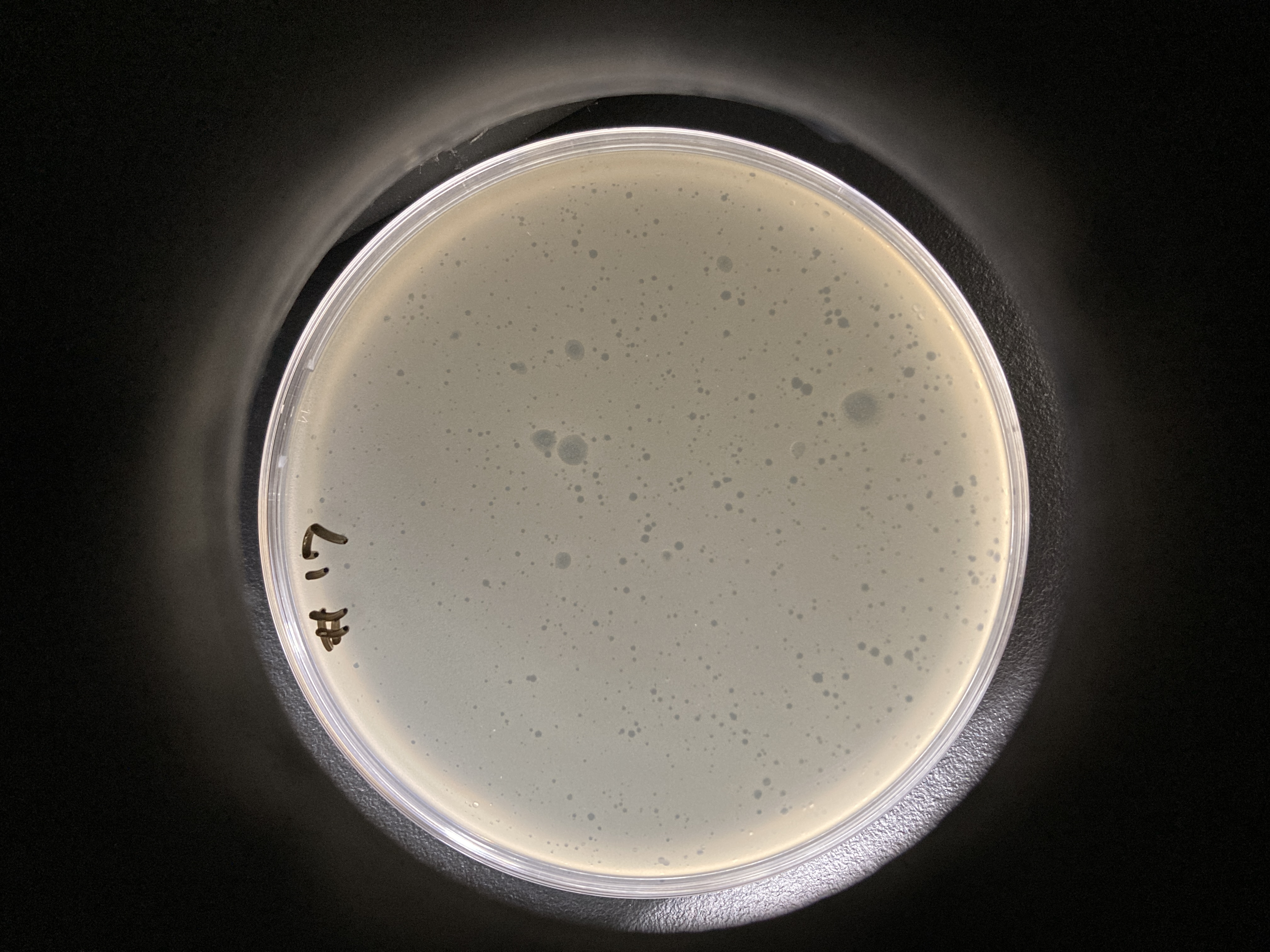

Supplement: Supplementary file 1 [file viruses-18-00092-s001.zip › No17.jpg]

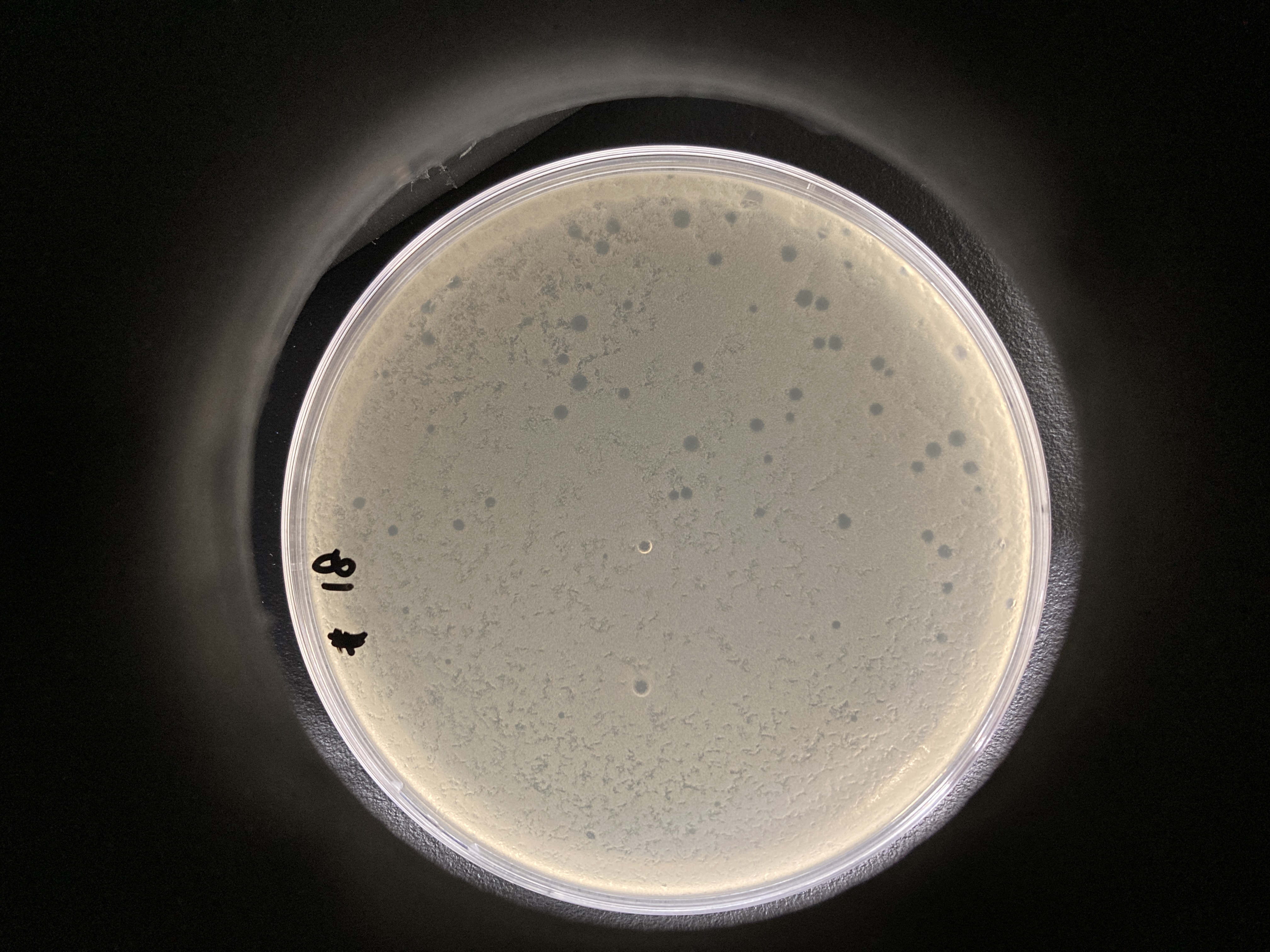

Supplement: Supplementary file 1 [file viruses-18-00092-s001.zip › No18.jpg]

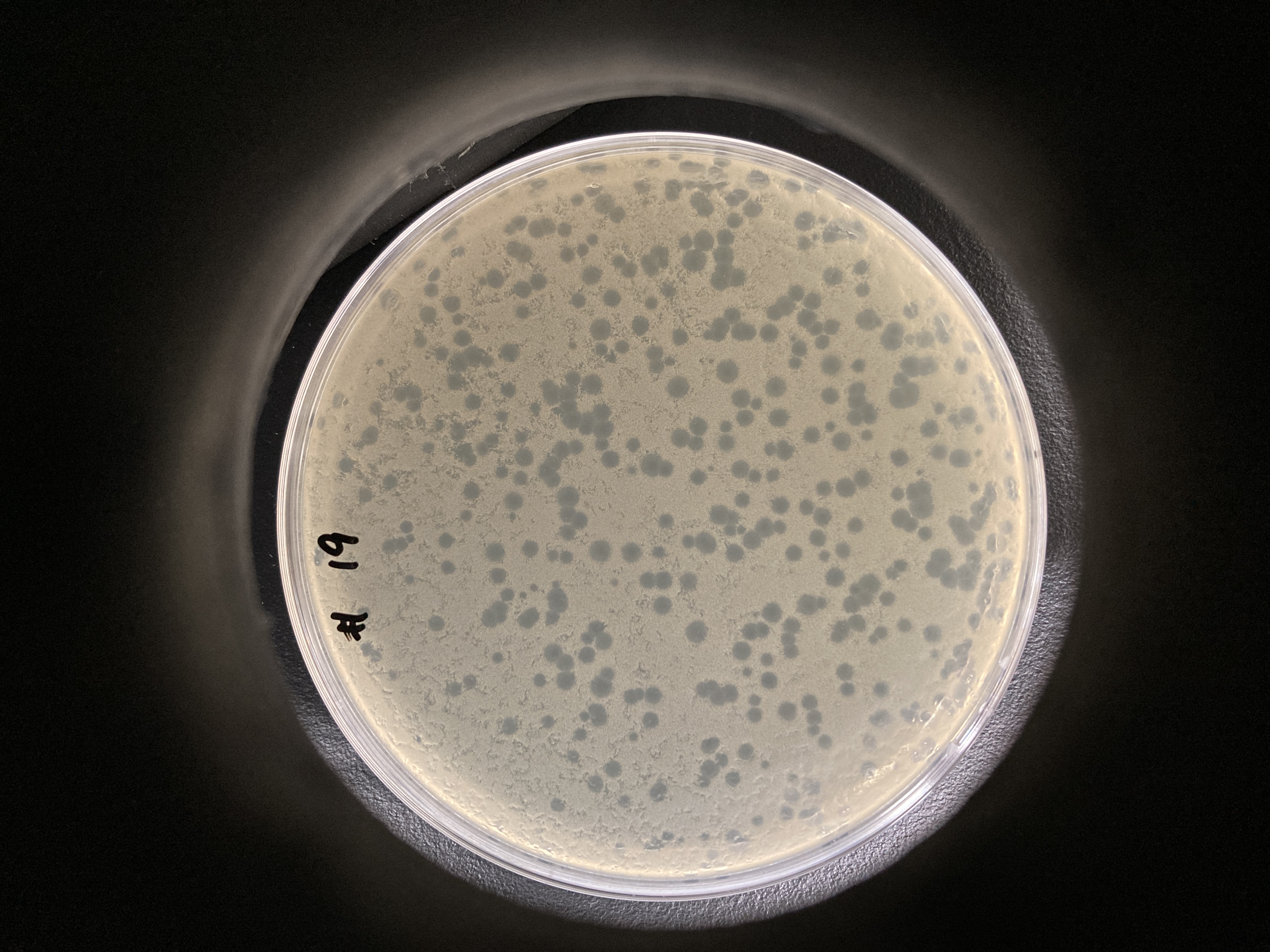

Supplement: Supplementary file 1 [file viruses-18-00092-s001.zip › No19.jpg]

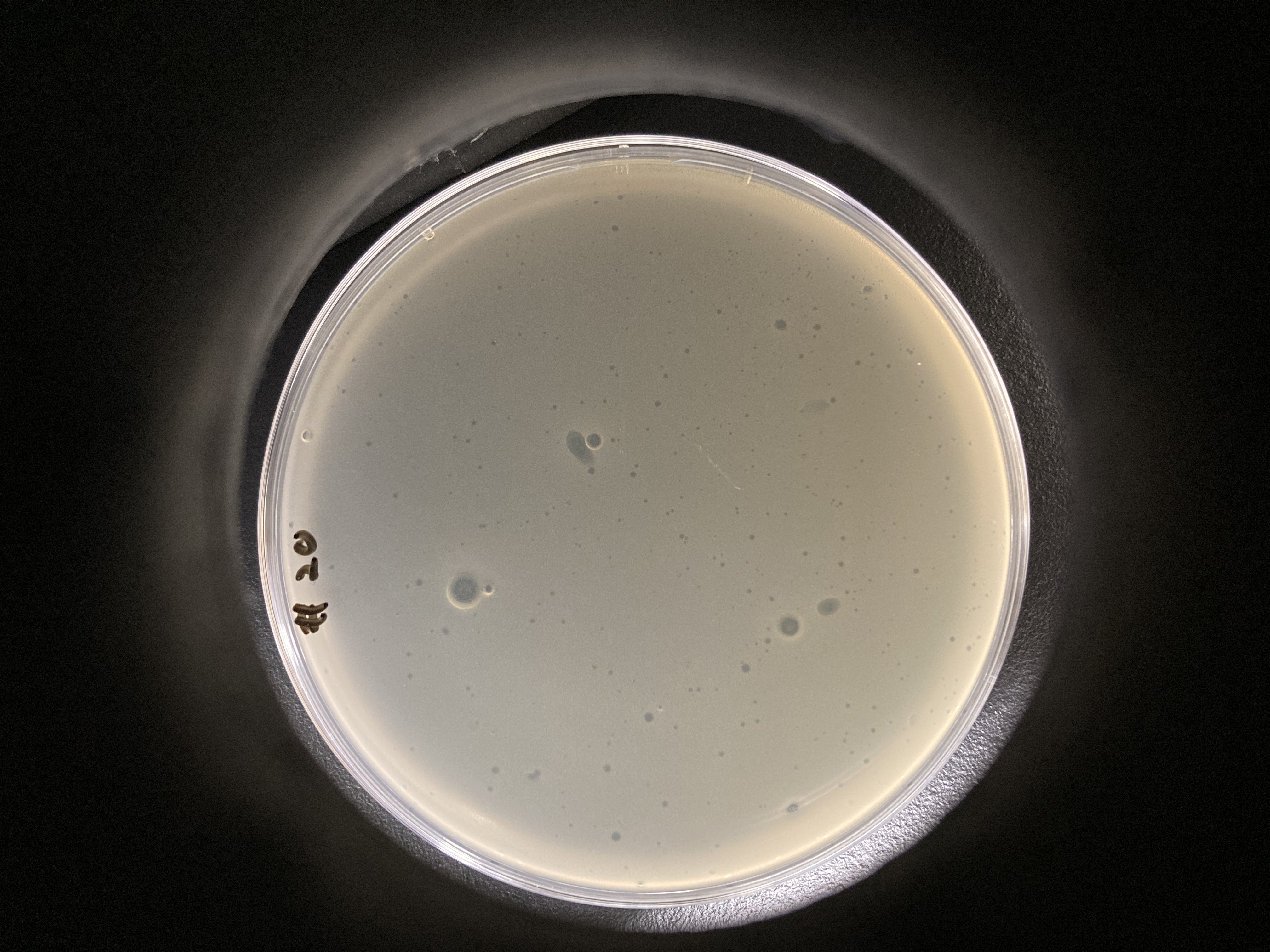

Supplement: Supplementary file 1 [file viruses-18-00092-s001.zip › No20.jpg]

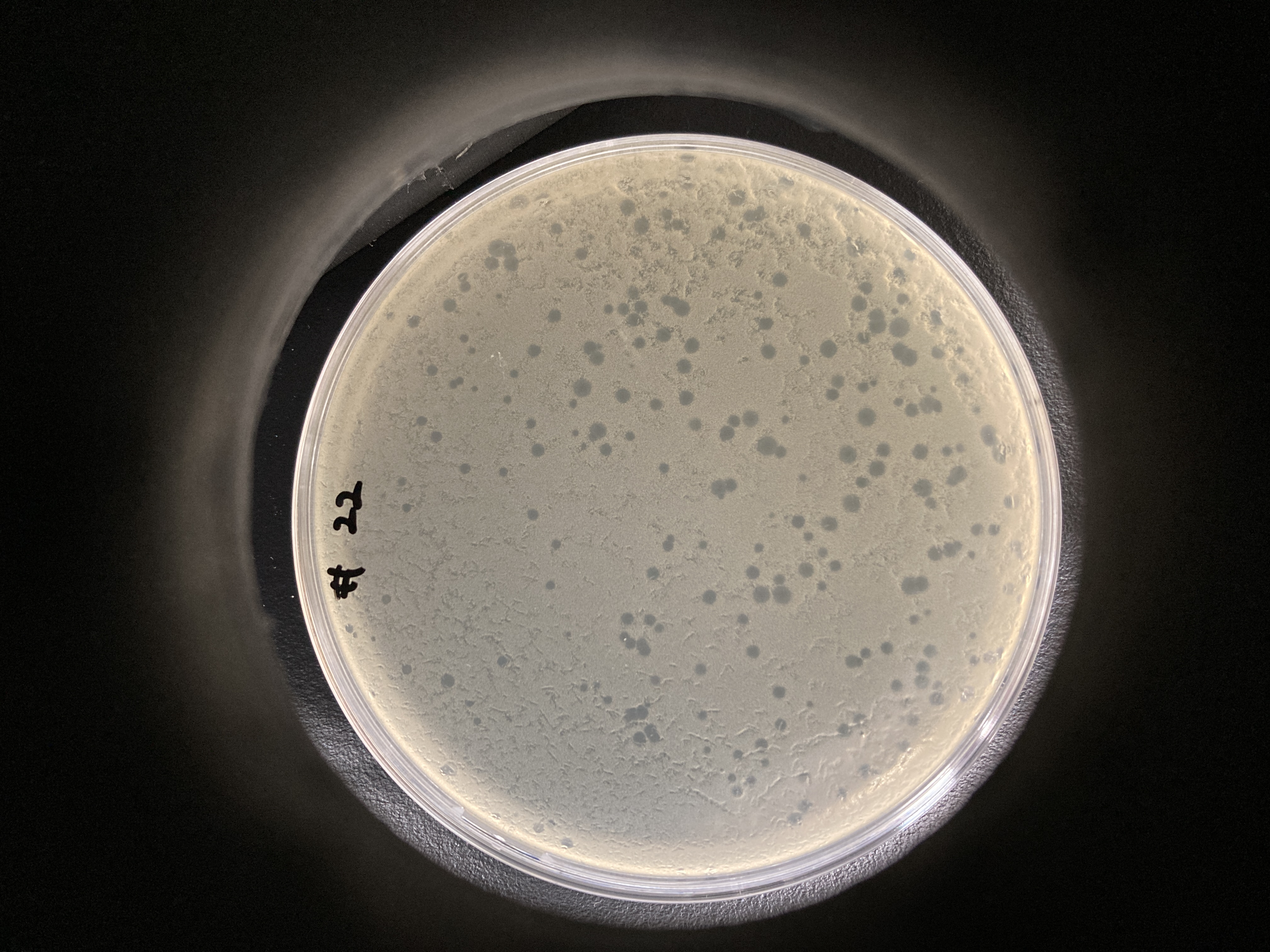

Supplement: Supplementary file 1 [file viruses-18-00092-s001.zip › No22.jpg]

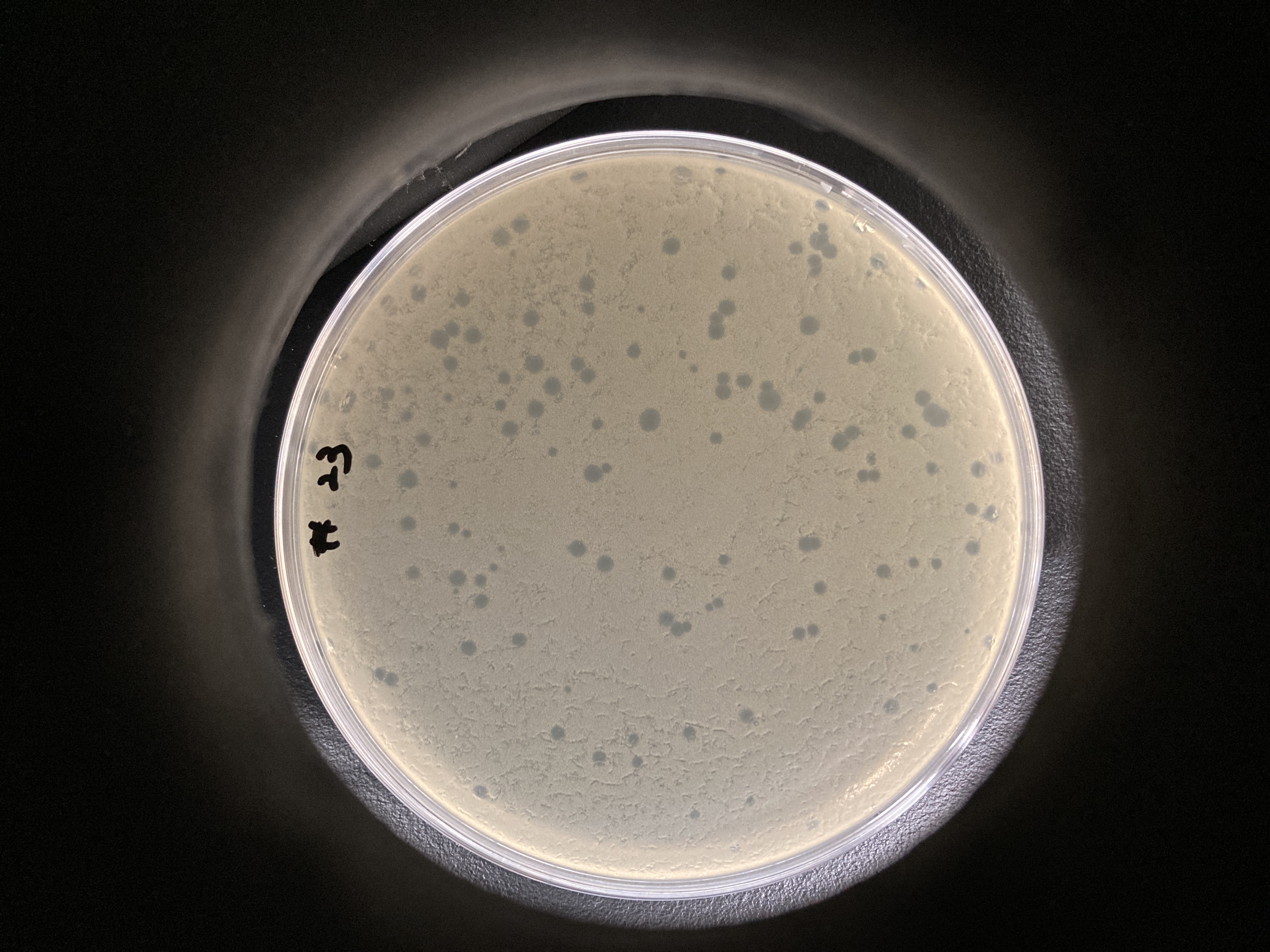

Supplement: Supplementary file 1 [file viruses-18-00092-s001.zip › No23.jpg]

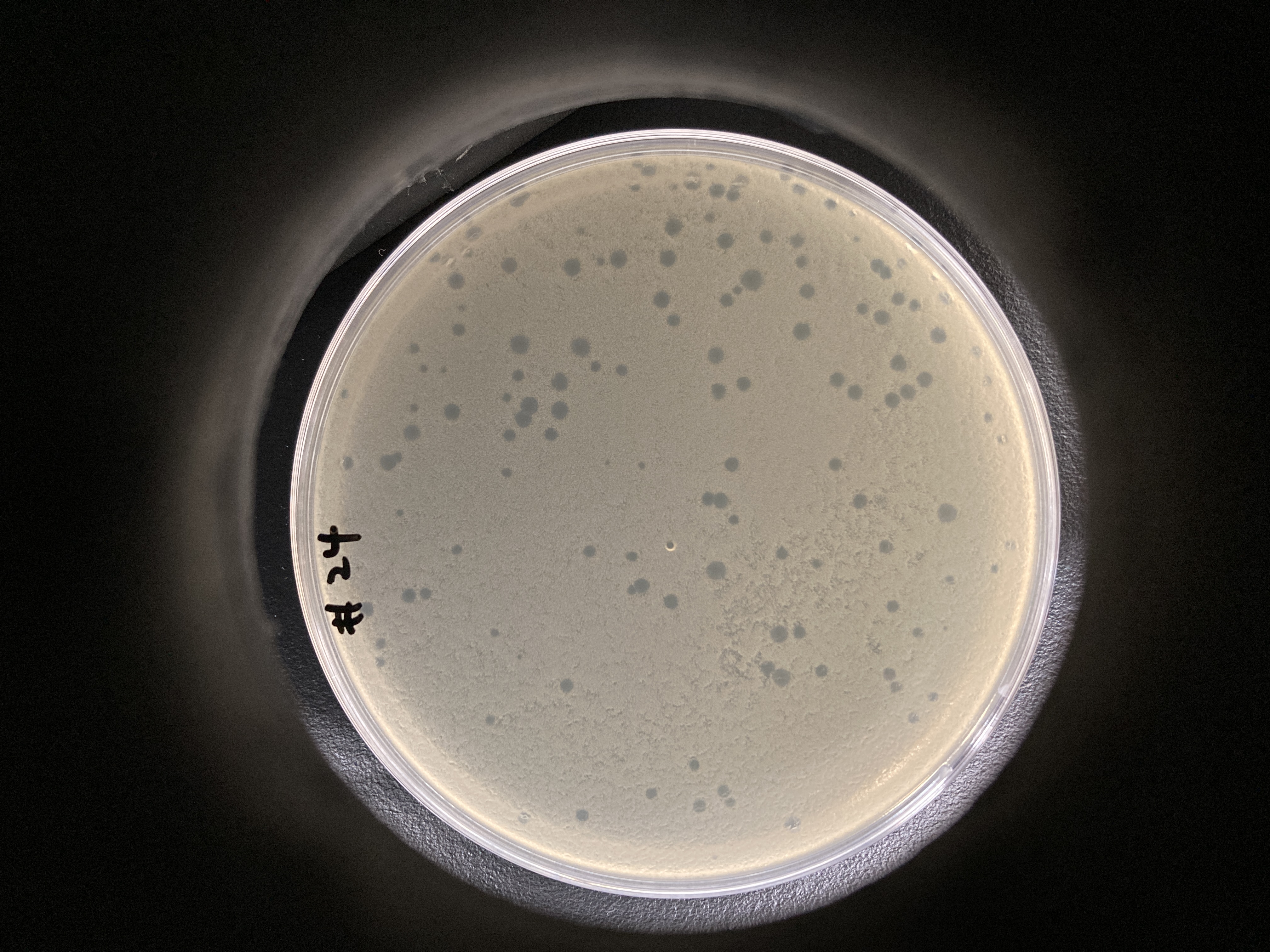

Supplement: Supplementary file 1 [file viruses-18-00092-s001.zip › No24.jpg]

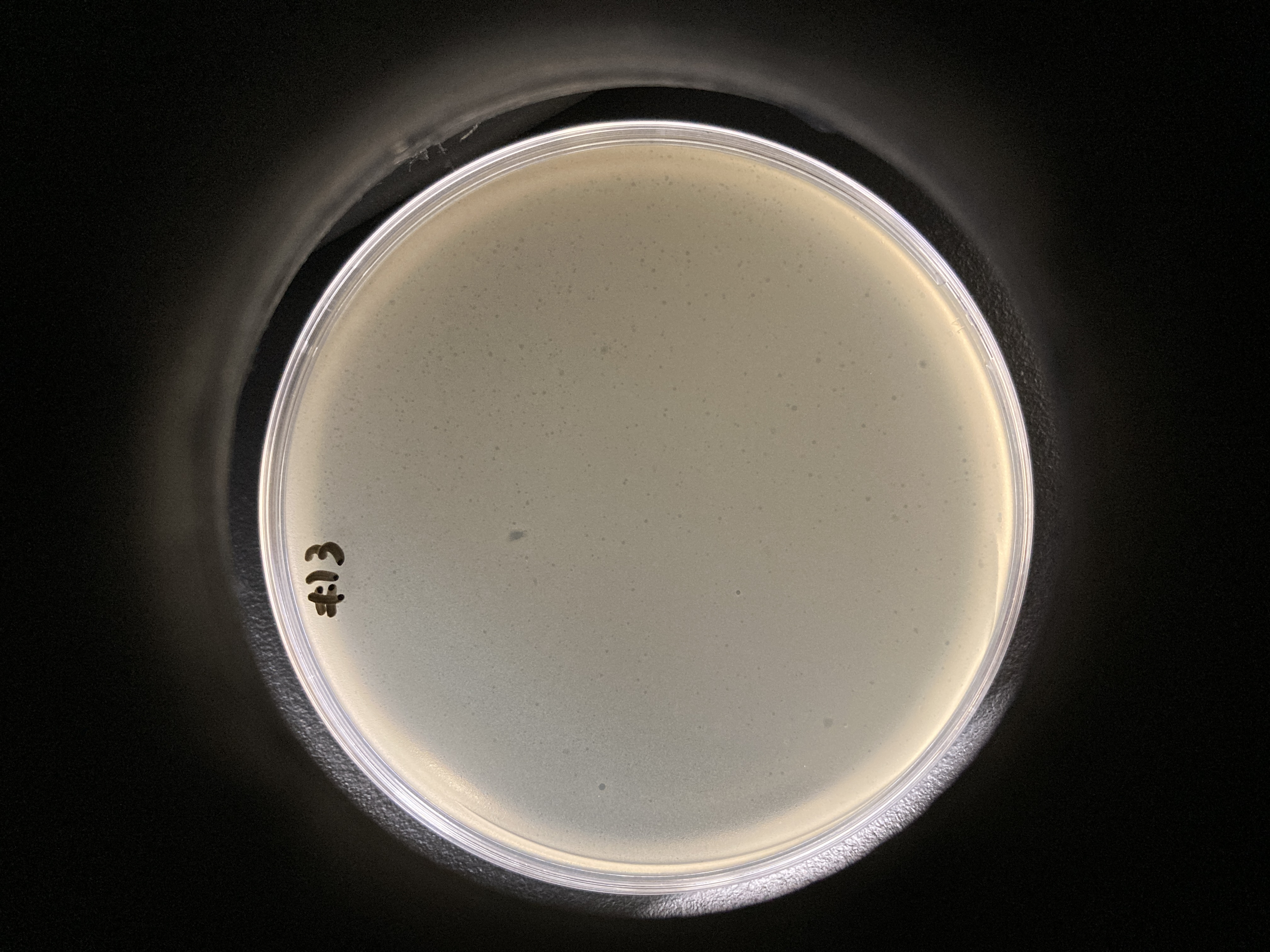

Supplement: Supplementary file 1 [file viruses-18-00092-s001.zip › No3.jpg]

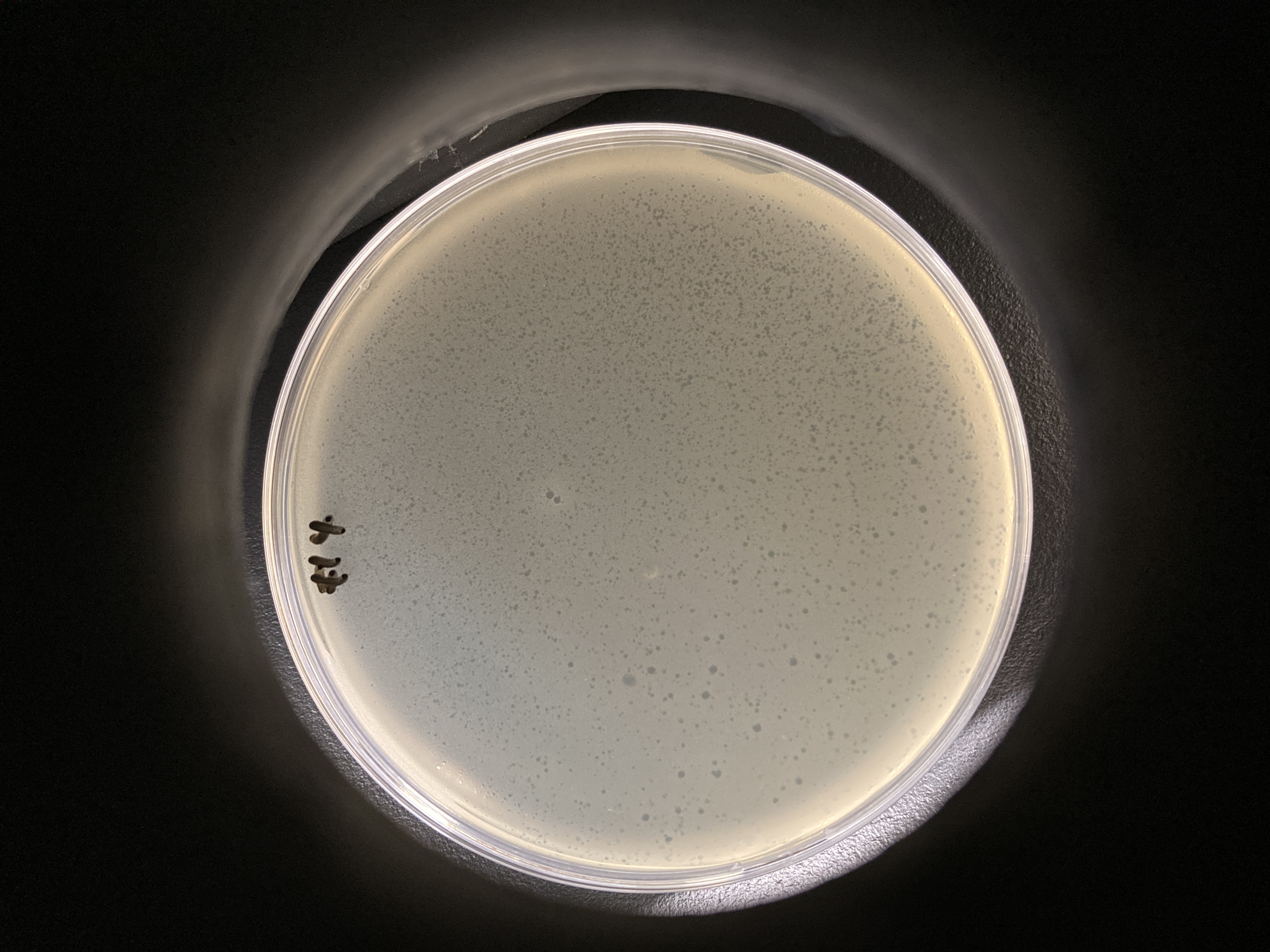

Supplement: Supplementary file 1 [file viruses-18-00092-s001.zip › No4.jpg]

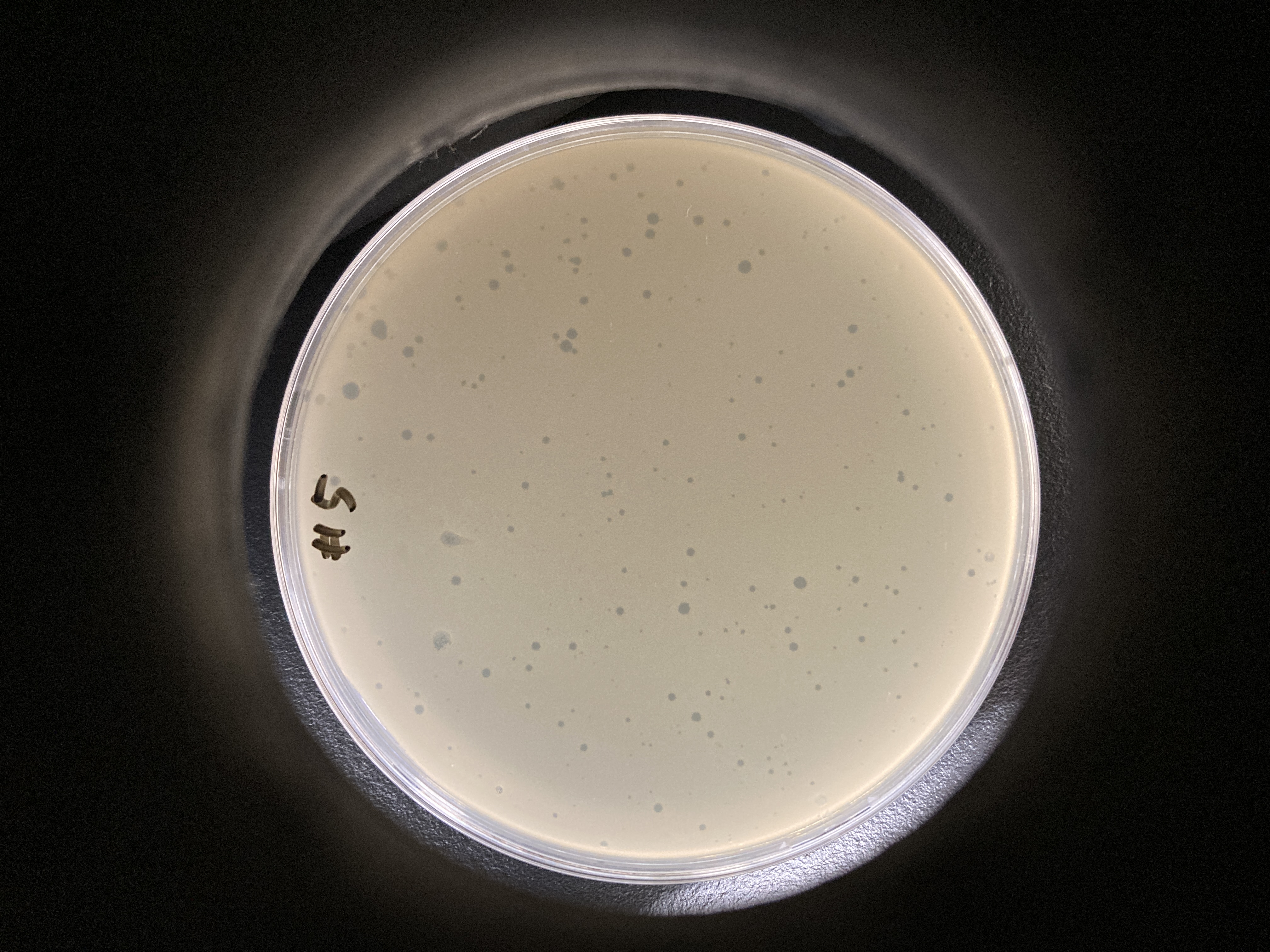

Supplement: Supplementary file 1 [file viruses-18-00092-s001.zip › No5.jpg]

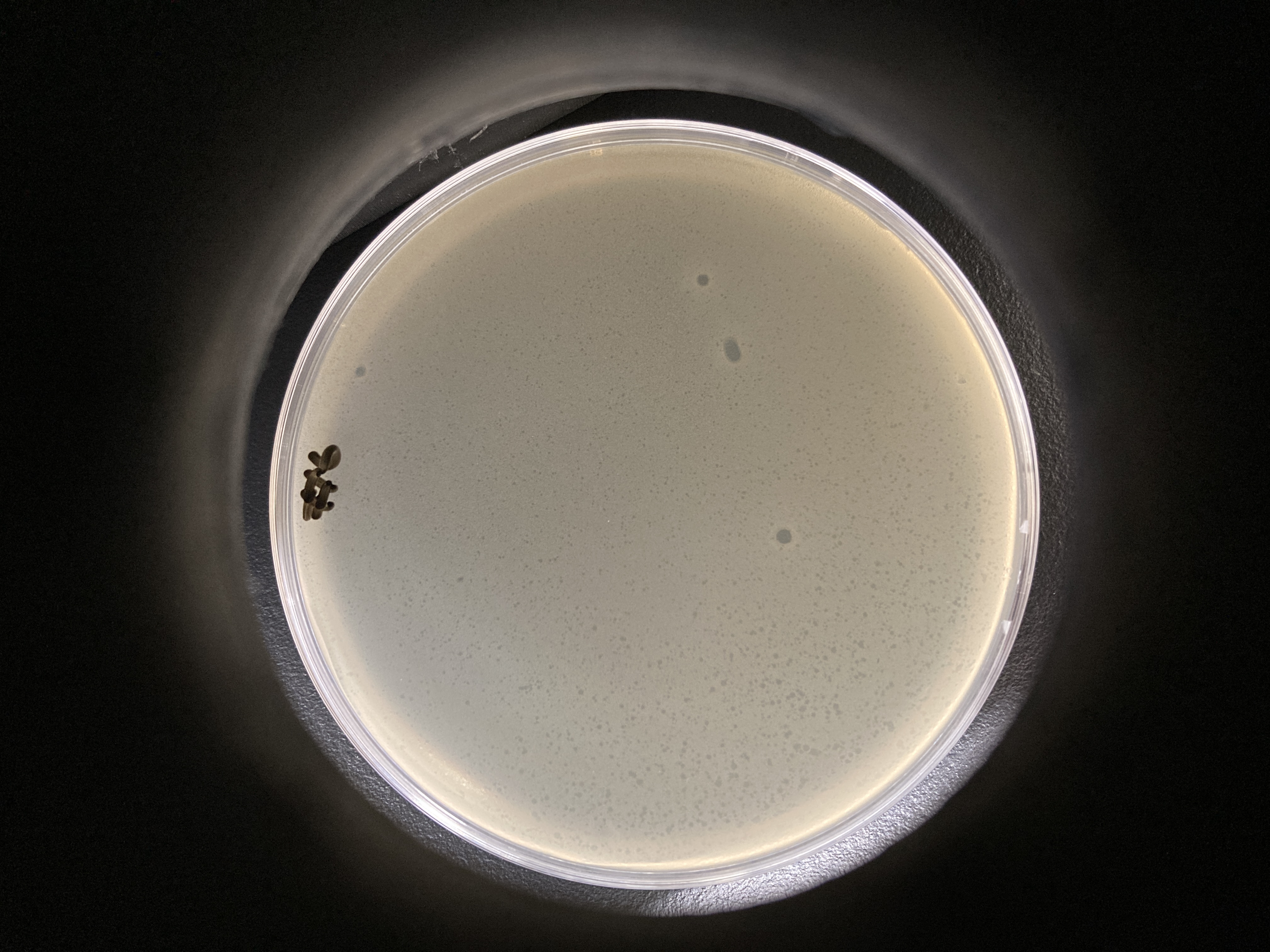

Supplement: Supplementary file 1 [file viruses-18-00092-s001.zip › No6.jpg]

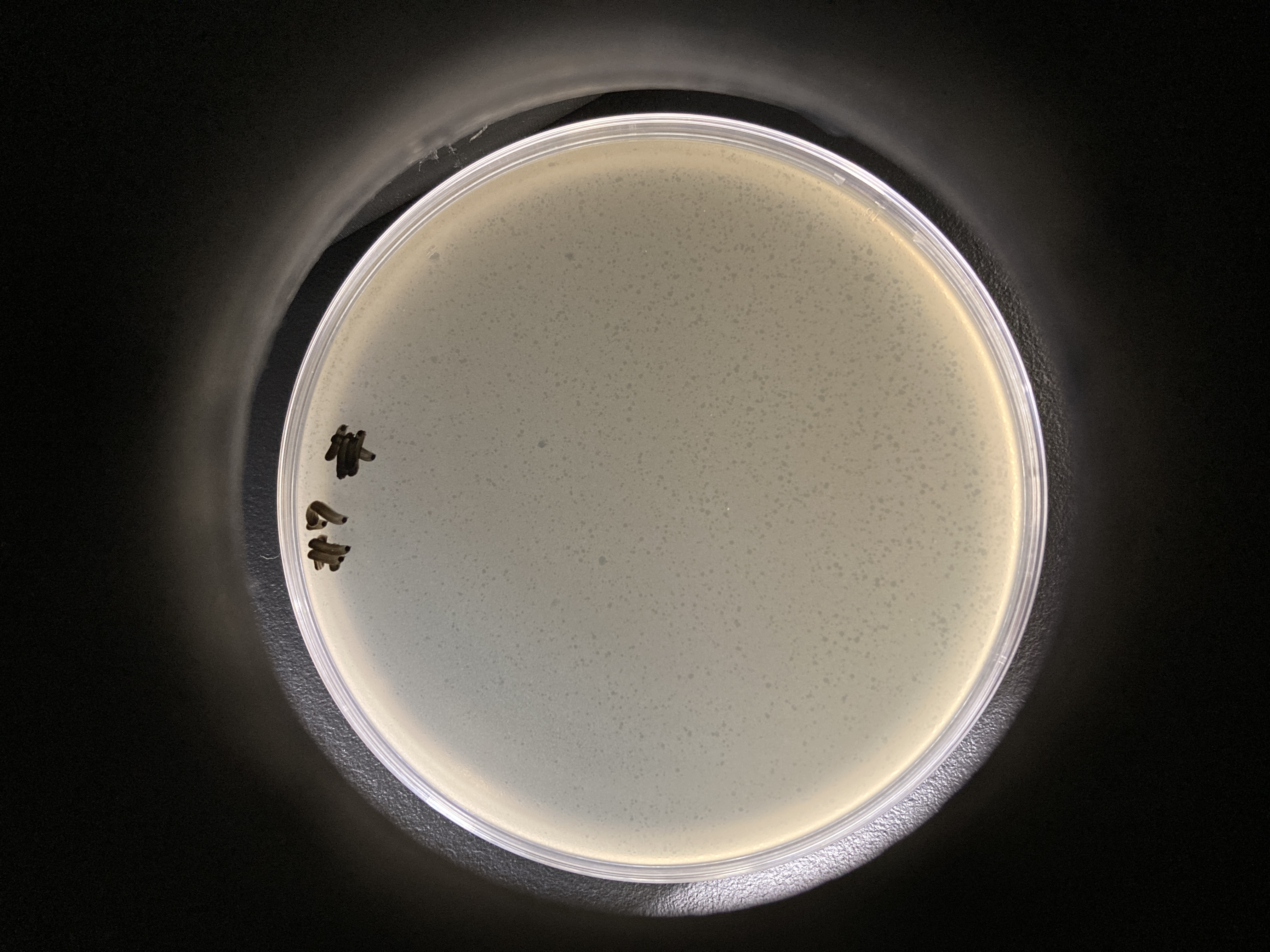

Supplement: Supplementary file 1 [file viruses-18-00092-s001.zip › No7.jpg]

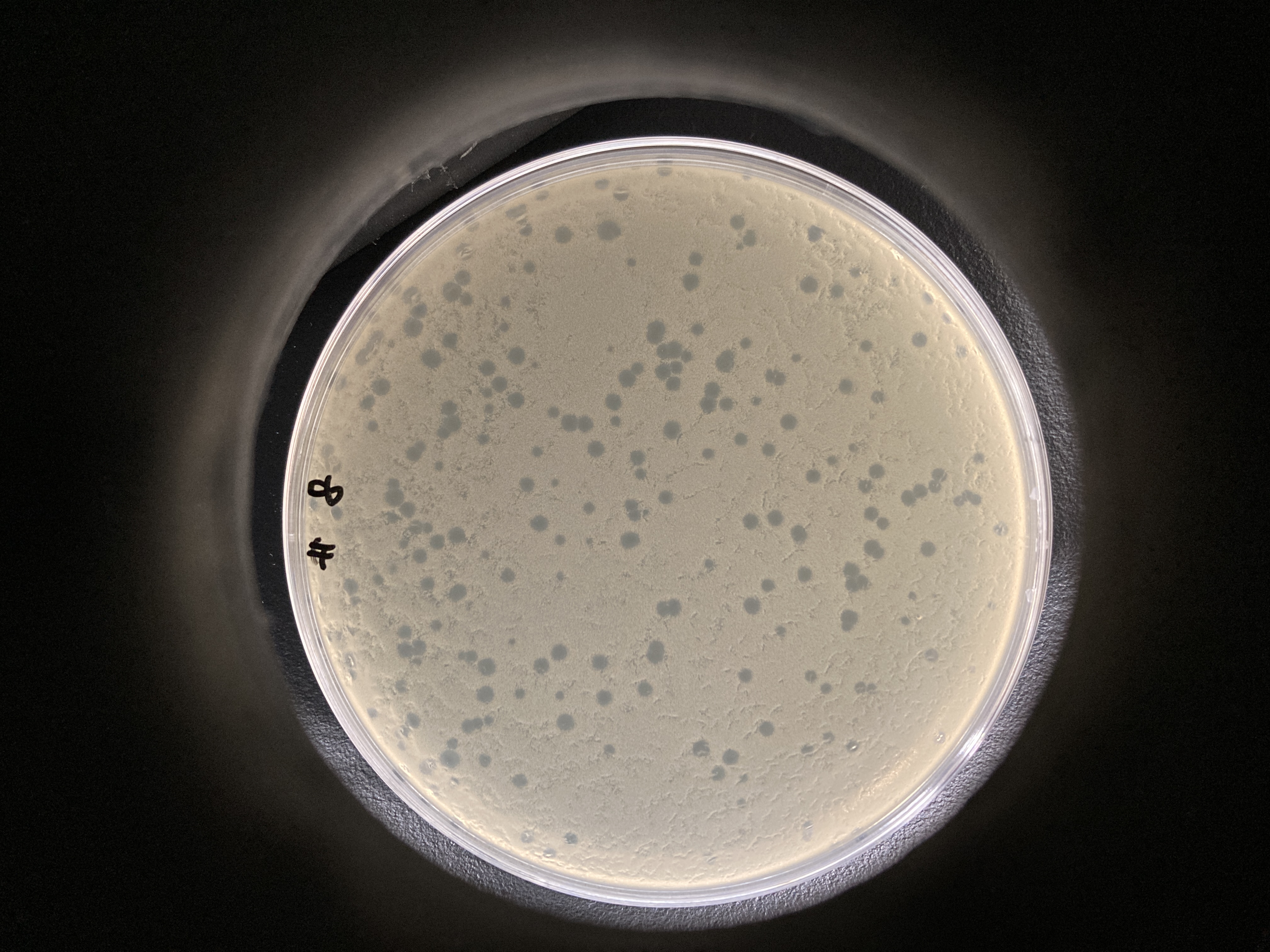

Supplement: Supplementary file 1 [file viruses-18-00092-s001.zip › No8.jpg]

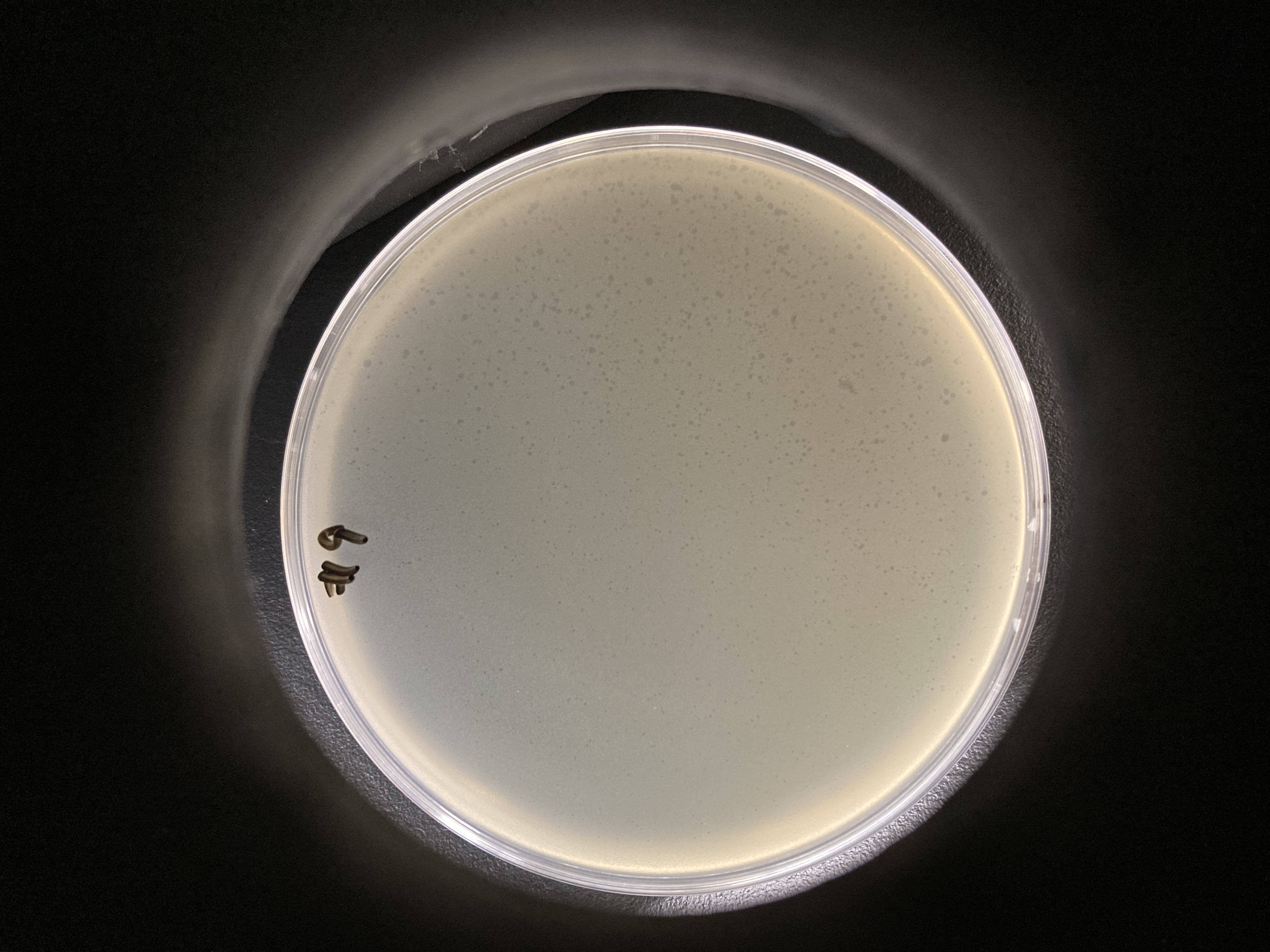

Supplement: Supplementary file 1 [file viruses-18-00092-s001.zip › No9.jpg]

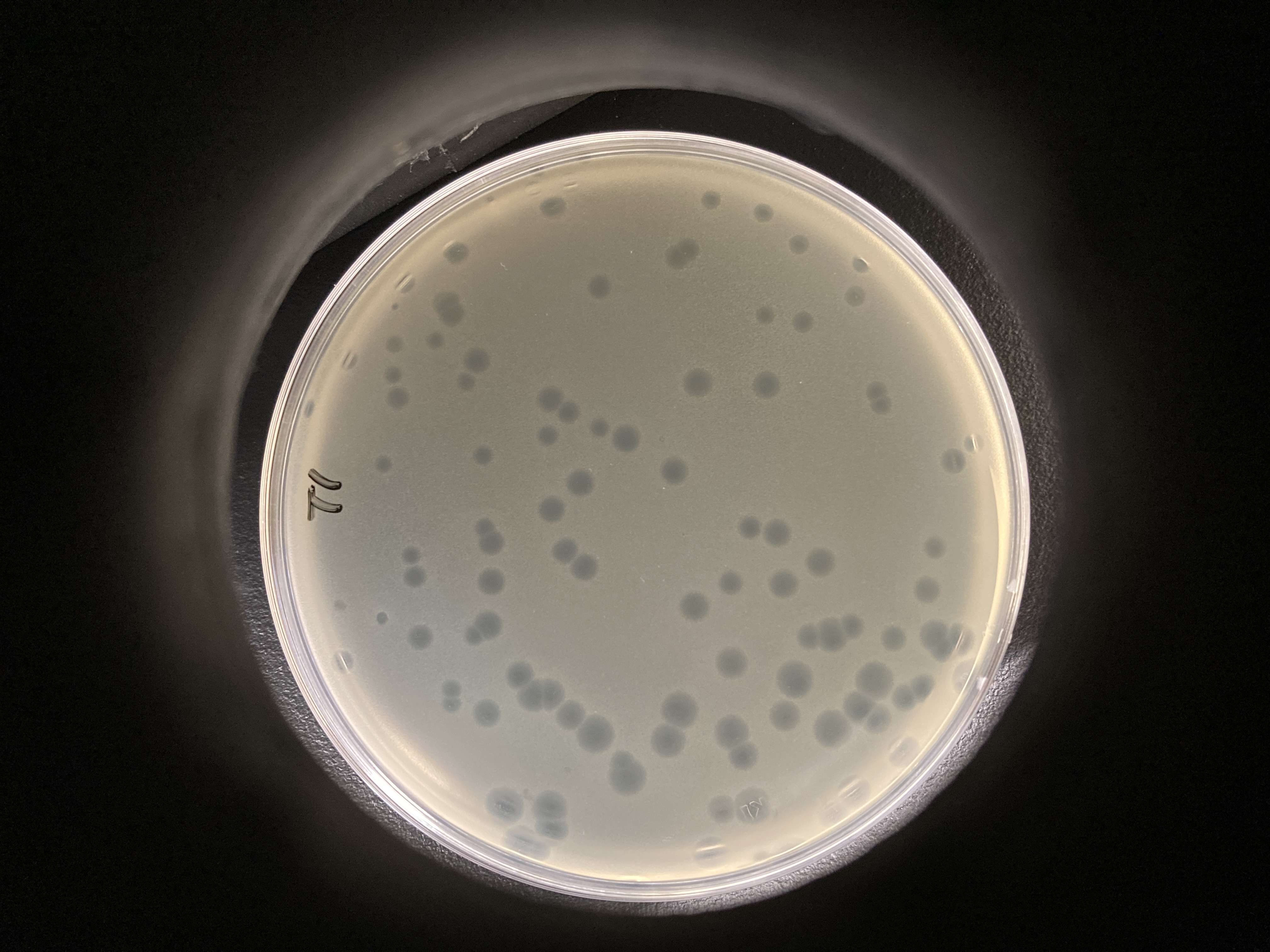

Supplement: Supplementary file 1 [file viruses-18-00092-s001.zip › T1.jpg]

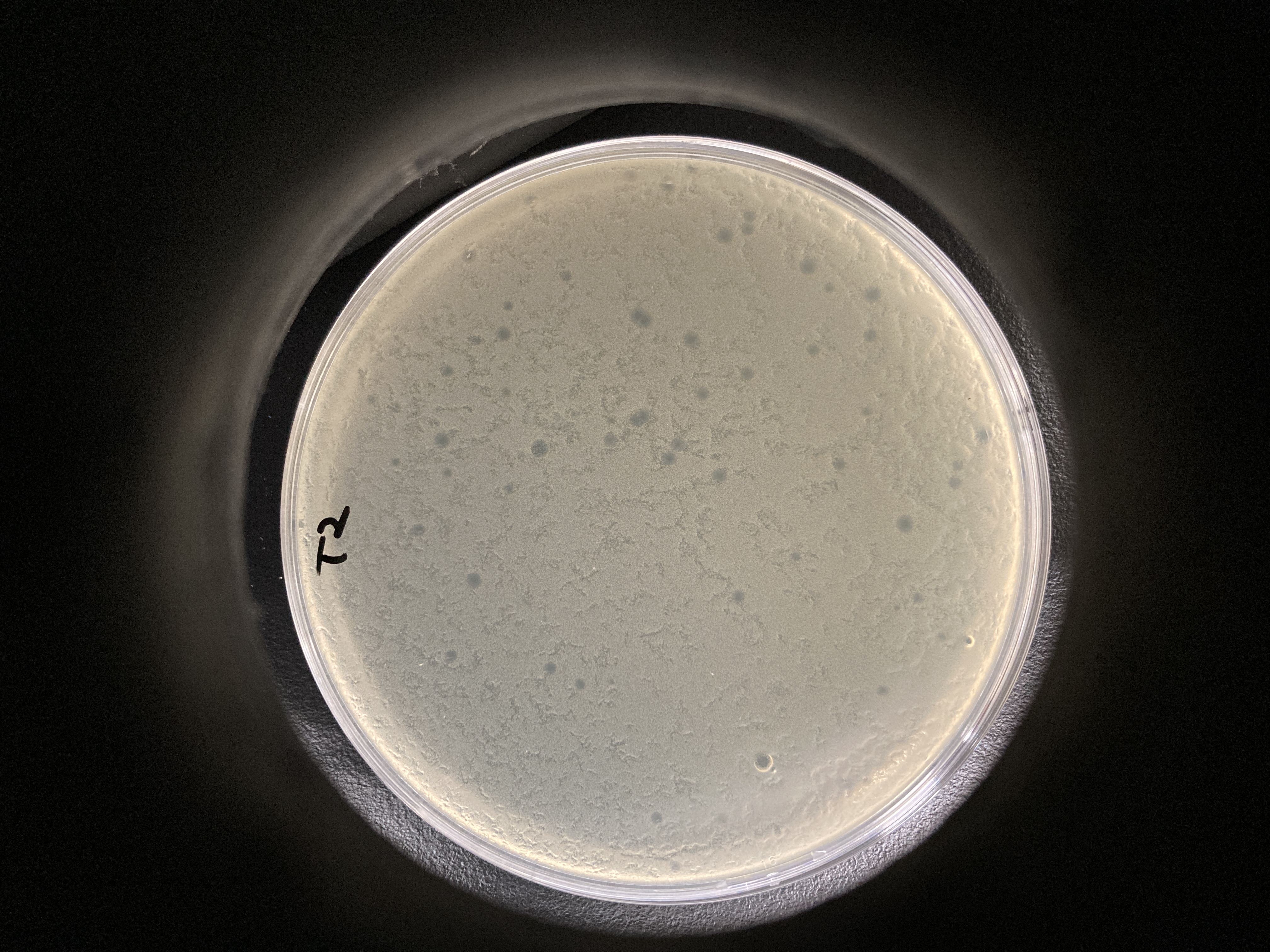

Supplement: Supplementary file 1 [file viruses-18-00092-s001.zip › T2.jpg]

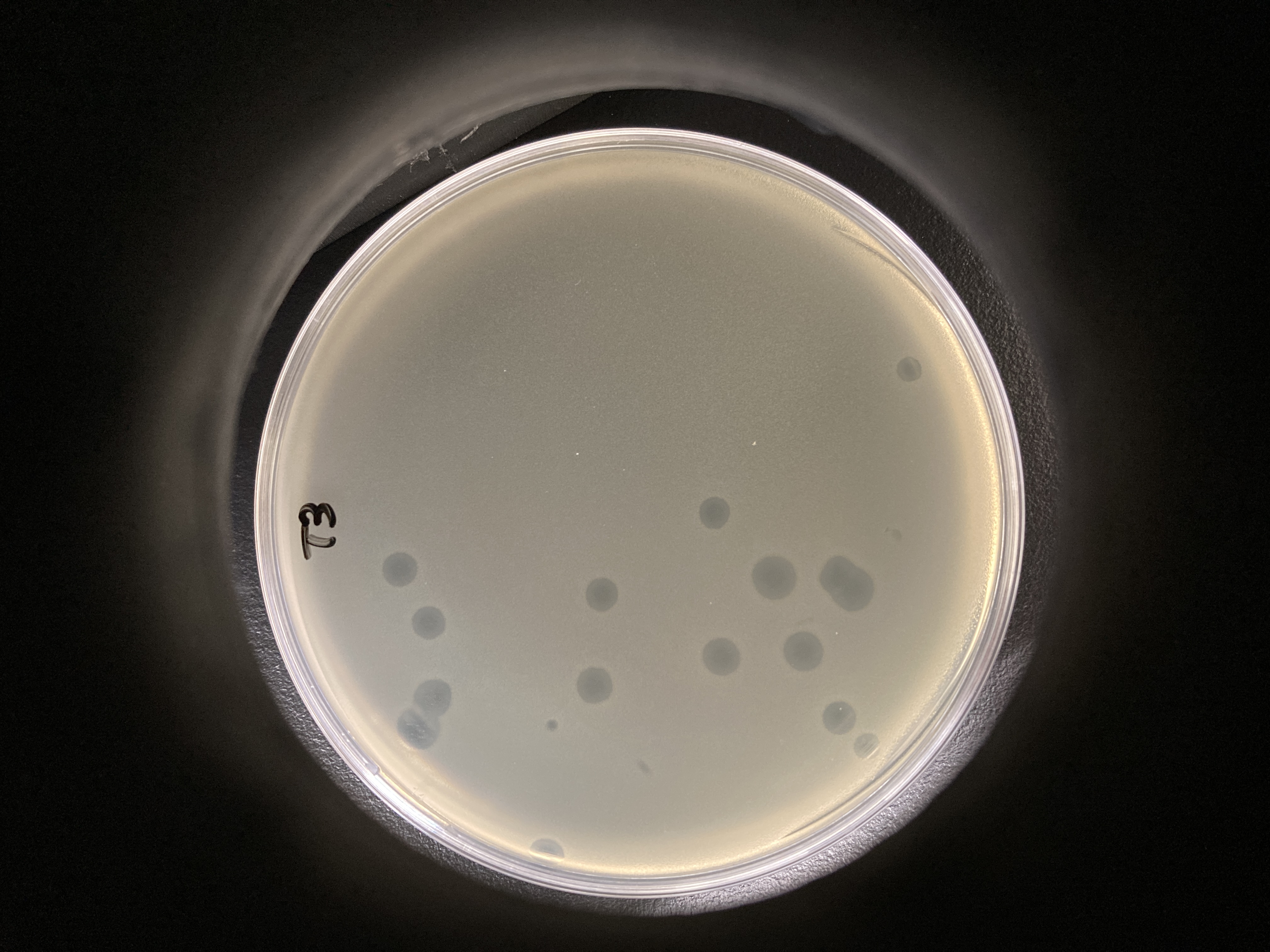

Supplement: Supplementary file 1 [file viruses-18-00092-s001.zip › T3.jpg]

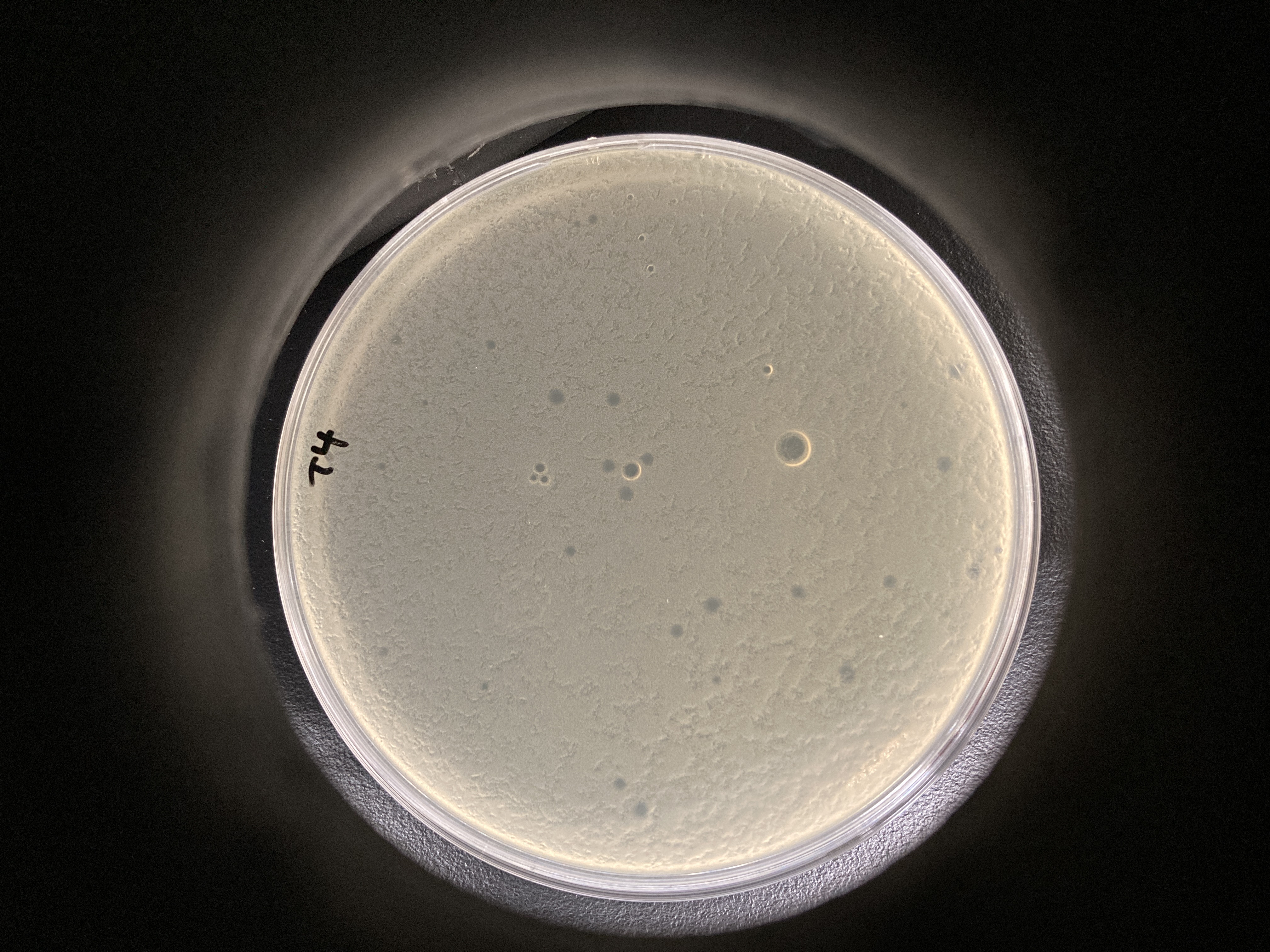

Supplement: Supplementary file 1 [file viruses-18-00092-s001.zip › T4.jpg]

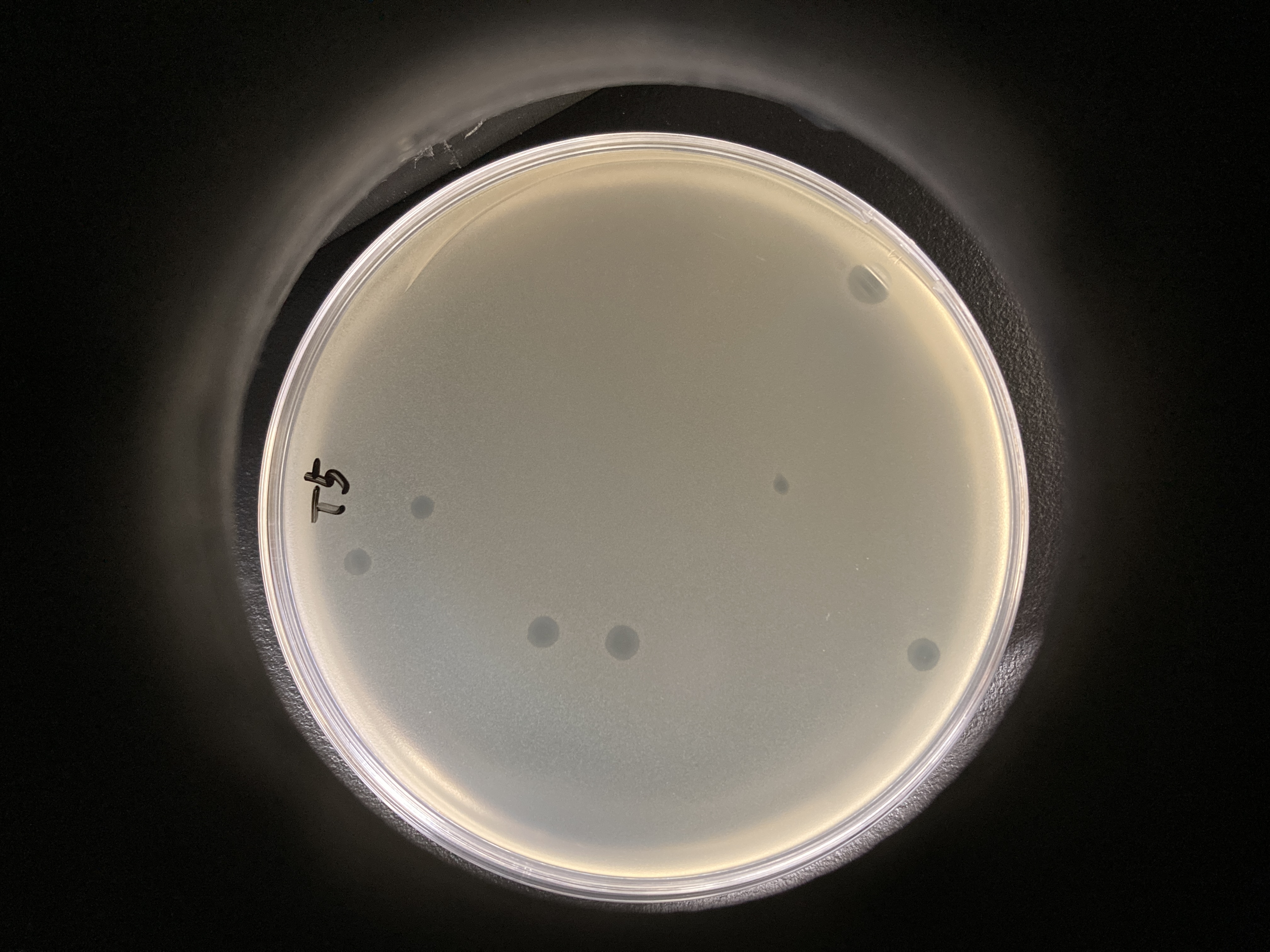

Supplement: Supplementary file 1 [file viruses-18-00092-s001.zip › T5.jpg]

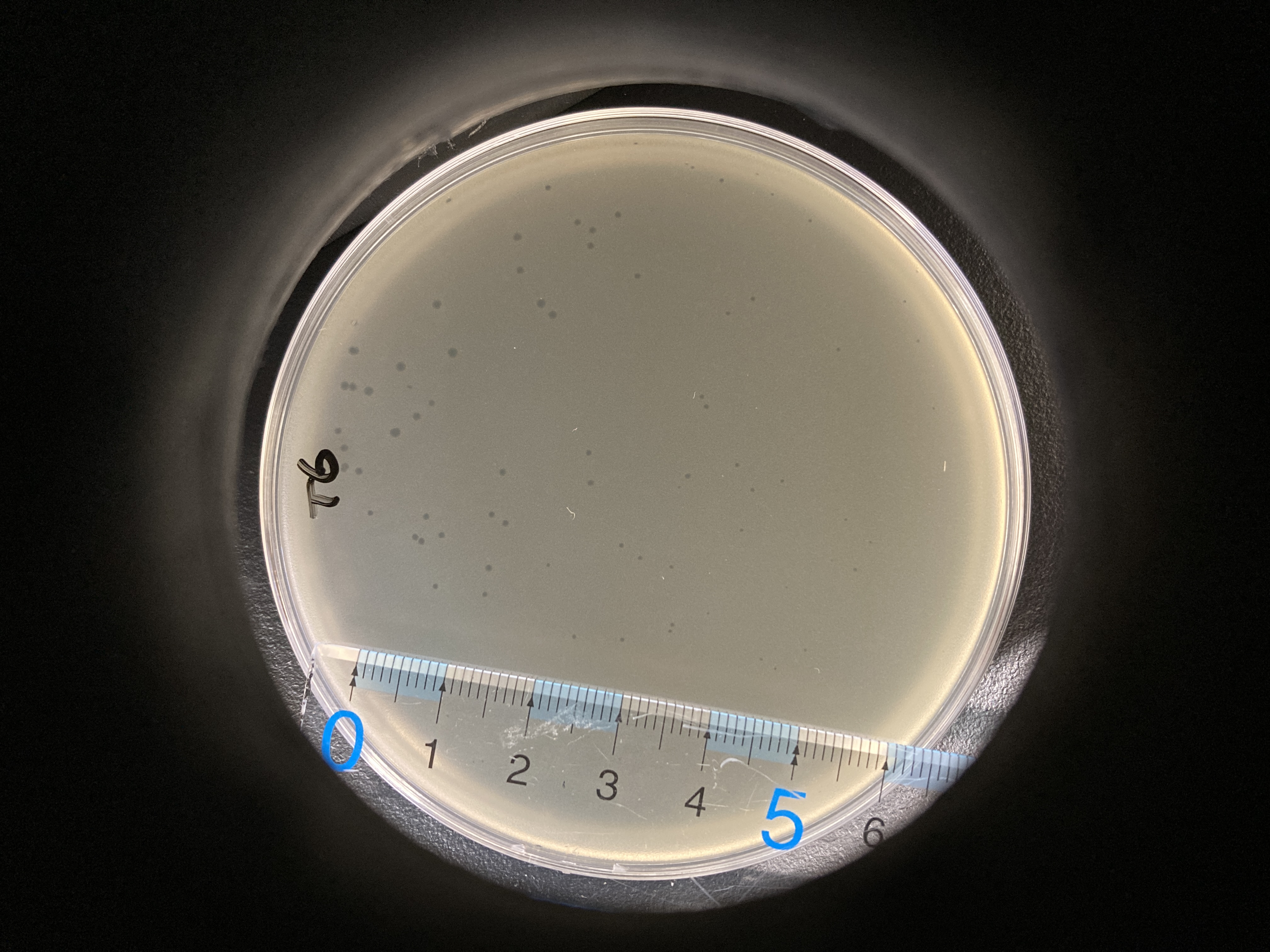

Supplement: Supplementary file 1 [file viruses-18-00092-s001.zip › T6.jpg]

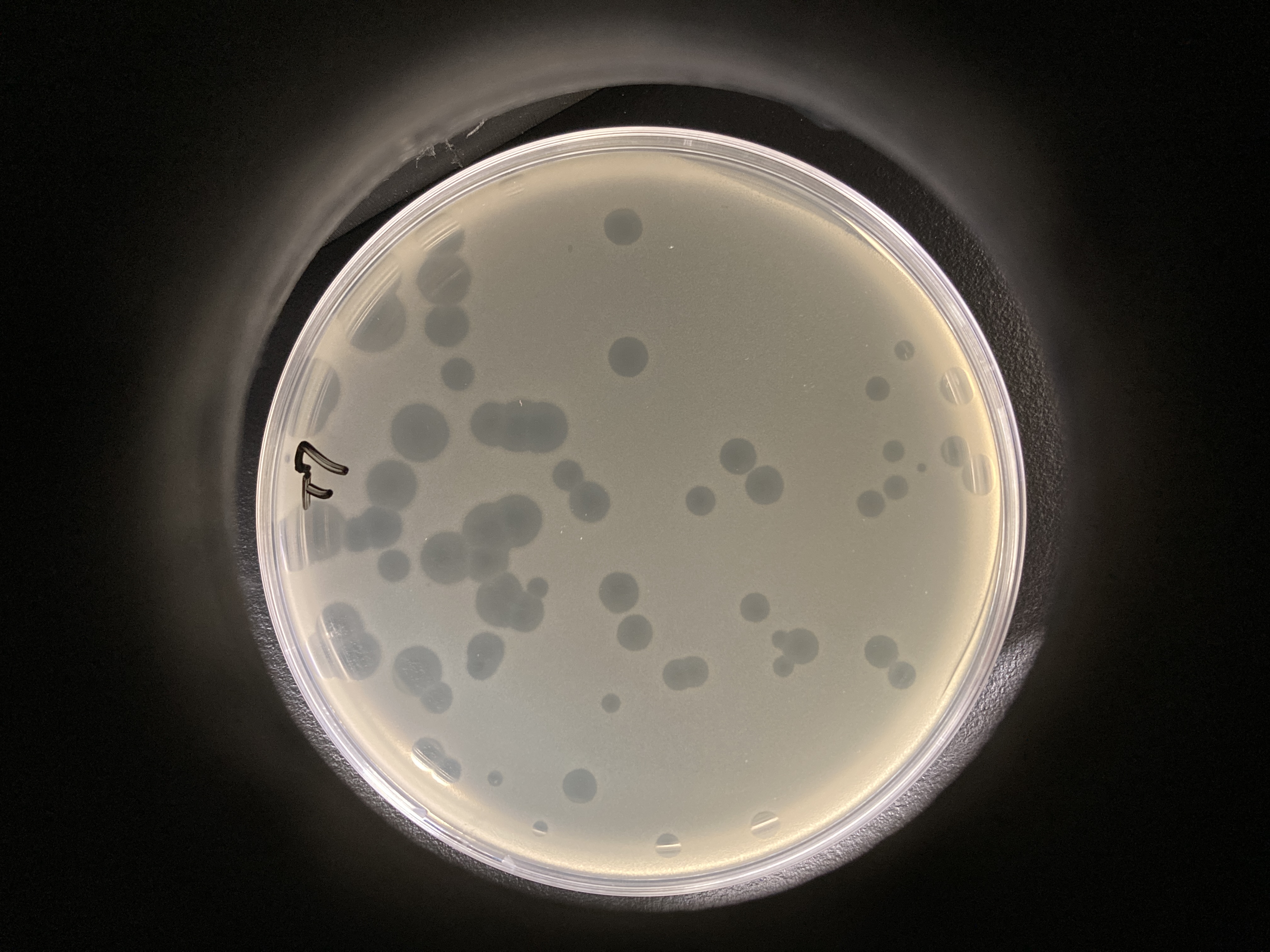

Supplement: Supplementary file 1 [file viruses-18-00092-s001.zip › T7.jpg]
